# Genetically predicted telomere length and the risk of 11 hematological diseases: a Mendelian randomization study

**DOI:** 10.18632/aging.205583

**Published:** 2024-02-22

**Authors:** Yimin Wang, Qi Liu, Shibing Liang, Minghao Yao, Huimin Zheng, Dongqing Hu, Yifei Wang

**Affiliations:** 1 The First Clinical Medical College, Shandong University of Traditional Chinese Medicine, Jinan, China; 2 Affiliated Hospital of Shandong University of Traditional Chinese Medicine, Jinan, China

**Keywords:** telomere length, hematologic diseases, Mendelian randomization, GWAS data, single nucleotide polymorphisms

## Abstract

Objective: Previous studies have demonstrated that various hematologic diseases (HDs) induce alterations in telomere length (TL). The aim of this study is to investigate whether genetically predicted changes in TL have an impact on the risk of developing HDs.

Methods: GWAS data for TL and 11 HDs were extracted from the database. The R software package “TwoSampleMR” was employed to conduct a two-sample Mendelian randomization (MR) analysis, in order to estimate the influence of TL changes on the risk of developing the 11 HDs.

Results: We examined the effect of TL changes on the risk of developing the 11 HDs. The IVW results revealed a significant causal association between genetically predicted longer TL and the risk of developing acute lymphocytic leukemia (ALL), acute myeloid leukemia (AML), chronic lymphocytic leukemia (CLL), mantle cell lymphoma (MANTLE), and hodgkin lymphoma (HODGKIN). However, there was no significant causal relationship observed between TL changes and the risk of developing chronic myeloid leukemia (CML), diffuse large b-cell lymphoma (DLBCL), marginal zone b-cell lymphoma (MARGINAL), follicular lymphoma (FOLLICULAR), monocytic leukemia (MONOCYTIC), and mature T/NK-cell lymphomas (TNK).

Conclusions: The MR analysis revealed a positive association between genetically predicted longer TL and an increased risk of developing ALL, AML, CLL, MANTLE, and HODGKIN. This study further supports the notion that cells with longer TL have greater proliferative and mutational potential, leading to an increased risk of certain HDs.

## INTRODUCTION

Telomeres served as protective structures located at the ends of chromosomes, and their gradual shortening was intricately linked to the occurrence and progression of cellular senescence, cancer, and various other diseases [1–3]. Recently, there has been extensive research on the role of telomeres in hematologic diseases (HDs). Telomeres have been found to be closely associated with hematopoietic stem cells (HSCs), hematological tumors, and complications arising from hematopoietic stem cell transplantation (HSCT) [4, 5].

Current research has mostly focused on the impact of telomere length (TL) changes on the progression and prognosis of various HDs. For instance, a study revealed that TL of HSCs was significantly reduced in patients with multiple myeloma, which was closely associated with disease progression and prognosis [6]. In patients with acute myeloid leukemia (AML), TL was significantly shortened and associated with disease development and therapeutic efficacy [7, 8]. Studies have shown that HSCT with longer TL has a higher success rate, leading to improved immune system reconstitution, enhanced anti-infection ability, and better anti-tumor effects [9]. In terms of treatment, therapeutic strategies targeting telomeres, such as telomerase inhibitors, telomere-lengthening agents, and the gene therapy, have shown promise in laboratory and clinical trials, providing novel avenues for HDs treatment [10, 11].

However, there is currently no systematic research on the impact of TL changes on the risk of developing various HDs. In recent years, some studies have utilized Mendelian randomization (MR) methods to predict the relationship between TL changes and the risk of developing various diseases. MR can mitigate the problem of reverse causality by using genetic variation as a natural randomization, thus excluding the impact of disease on TL and yielding more reliable conclusions. It can uncover novel causal relationships, such as the causal link between TL and chronic obstructive pulmonary disease [12], Alzheimer’s disease [13], cancer [14], and other diseases, thereby providing new insights for the prevention and treatment of related conditions. Based on this, we aim to leverage the unique advantages of using MR for telomere research to systematically elucidate the impact of genetically predicted TL changes on the risk of developing various HDs.

## MATERIALS AND METHODS

### Study design description

**Figure 1:**
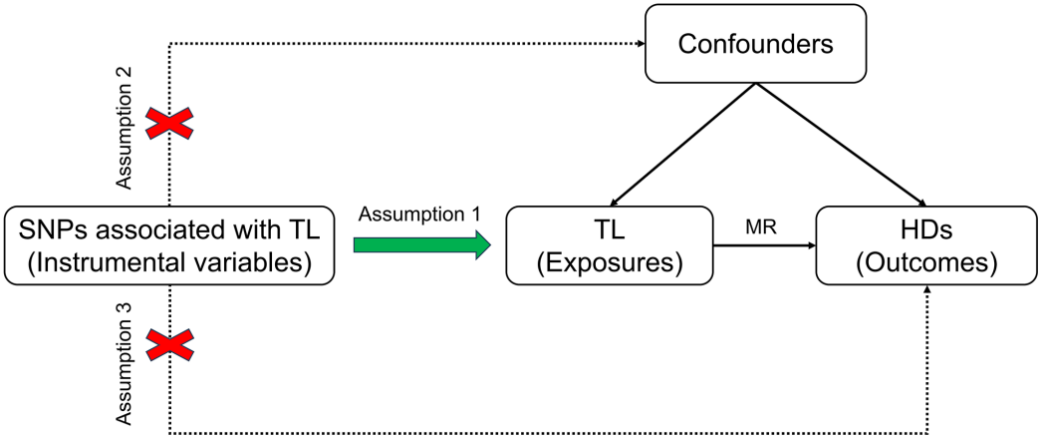
**Schematic diagram illustrating the MR.** Abbreviations: SNP: single nucleotide polymorphism; TL: telomere length; MR: Mendelian randomization; HDs: hematologic diseases.

The design of the MR study must adhere to three fundamental assumptions: 1) a strong correlation between instrumental variables (IVs) and exposure factors; 2) confounding factors that are independent of the exposure-outcome relationship; 3) genetic variables that only influence outcomes through exposure, rather than through other means [15]. MR analysis was conducted using GWAS data, with TL considered as the exposure and 11 HDs as the outcomes to analyze their association. Figure 1 depicts the overall study design.

### Instrumental variables selection

The TL data were obtained from the UK Biobank (download address: https://gwas.mrcieu.ac.uk/datasets/ieu-b-4879/), including 472174 UKB participants [16]. The screening method employed for IVs was based on previous researchers’ approach [17]. Firstly, single nucleotide polymorphisms (SNPs) were selected at the threshold of genome-wide significance (*p* < 5 × 10^−8^). Secondly, the parameter r2 threshold was set to 0.001, the kilobase pair (kb) to 10000 and the LD clumping function was used to exclude interference in linkage disequilibrium. Thirdly, we queried all SNPs in PhenoScanner (http://www.phenoscanner.medschl.cam.ac.uk/) to ascertain if there were any SNPs associated with potential confounding factors or outcomes. Subsequently, the F statistic was employed to assess the strength of the IVs, with those having F < 10 being excluded. Lastly, we harmonized exposure and outcome data, excluding palindromic SNPs with intermediate allele frequencies. A total of 152 SNPs were selected for MR analysis (Supplementary File 1).

### Hematologic diseases data sources

The GWAS datasets for the 11 HDs (Table 1) were obtained from the FinnGen website (R10 version) (https://www.finngen.fi/en/). The GWAS data related to HDs included acute lymphocytic leukemia (ALL), acute myeloid leukemia (AML), chronic lymphocytic leukemia (CLL), chronic myeloid leukemia (CML), diffuse large b-cell lymphoma (DLBCL), follicular lymphoma (FOLLICULAR), Hodgkin lymphoma (HODGKIN), mantle cell lymphoma (MANTLE), marginal zone b-cell lymphoma (MARGINAL), monocytic leukemia (MONOCYTIC), and mature T/NK-cell lymphomas (TNK). As the aforementioned GWAS datasets were publicly available, ethical approval was not required.

**Table 1:** Hematologic diseases data sources.

**Diseases**	**Finngen ID**	**Population**	**SNPs**	**Cases**	**Controls**
ALL	finngen_R10_C3_ALL_EXALLC	Europeans	19680787	197	314192
AML	finngen_R10_C3_AML_EXALLC	Europeans	19680799	244	314192
CLL	finngen_R10_C3_CLL_EXALLC	Europeans	19680807	668	314189
CML	finngen_R10_C3_CML_EXALLC	Europeans	19050426	115	314192
DLBCL	finngen_R10_C3_DLBCL_EXALLC	Europeans	19680818	1050	314193
FOLLICULAR	finngen_R10_CD2_FOLLICULAR_LYMPHOMA_EXALLC	Europeans	19681177	1181	324650
HODGKIN	finngen_R10_CD2_HODGKIN_LYMPHOMA_EXALLC	Europeans	19681163	846	324650
MANTLE	finngen_R10_C3_MANTLE_CELL_LYMPHOMA_EXALLC	Europeans	19680787	210	314193
MARGINAL	finngen_R10_C3_MARGINAL_ZONE_LYMPHOMA_EXALLC	Europeans	19680791	202	314193
MONOCYTIC	finngen_R10_CD2_MONOCYTIC_LEUKAEMIA_EXALLC	Europeans	19681137	85	324650
TNK	finngen_R10_CD2_TNK_LYMPHOMA_EXALLC	Europeans	19681156	363	324650

Abbreviations: SNP: single nucleotide polymorphism; ALL: acute lymphocytic leukemia; AML: acute myeloid leukemia; CLL: chronic lymphocytic leukemia; CML: chronic myeloid leukemia; DLBCL: diffuse large b-cell lymphoma; FOLLICULAR: follicular lymphoma; HODGKIN: Hodgkin lymphoma; MANTLE: mantle cell lymphoma; MARGINAL: marginal zone b-cell lymphoma; MONOCYTIC: monocytic leukemia; TNK: mature T/NK-cell lymphomas.

### Statistical analysis

Six MR methods, namely inverse variance weighted (random effects) (IVW-RE), IVW (fixed effects) (IVW-FE), Weighted median, MR Egger, Simple mode, and Weighted mode were employed to estimate the relationship between TL and 11 HDs. In cases where the causal effect estimates from the six models were inconsistent, the results from the IVW-RE or IVW-FE method were considered as the primary outcome. The odds ratio (OR) and the corresponding 95% confidence interval (CI) were used to estimate the degree of causality, with OR>1 indicating exposure as a risk factor for the outcome, OR<1 indicating exposure as a protective factor for the outcome, and *p* < 0.05 indicating statistically significant causality. The Cochran’s *Q* test was utilized to detect heterogeneity between IVs, with *p* < 0.05 indicating heterogeneity. When there is no heterogeneity, IVW-FE is chosen; when heterogeneity exists, IVW-RE is chosen [18]. Sensitivity analysis was mainly conducted using the leave-one-out method, whereby one SNP was removed at a time to assess the stability and reliability of the causal relationship between the remaining SNPs and the outcome. An MR Egger intercept test was performed to detect the presence of directional pleiotropy in the IVs, with *p* < 0.05 indicating directional pleiotropy. Funnel plots were generated to assess directional pleiotropy. The MR analysis was implemented in R software (version 4.3.1) using the R package “TwoSampleMR” (version 0.5.6).

## RESULTS

**Figure 2:**
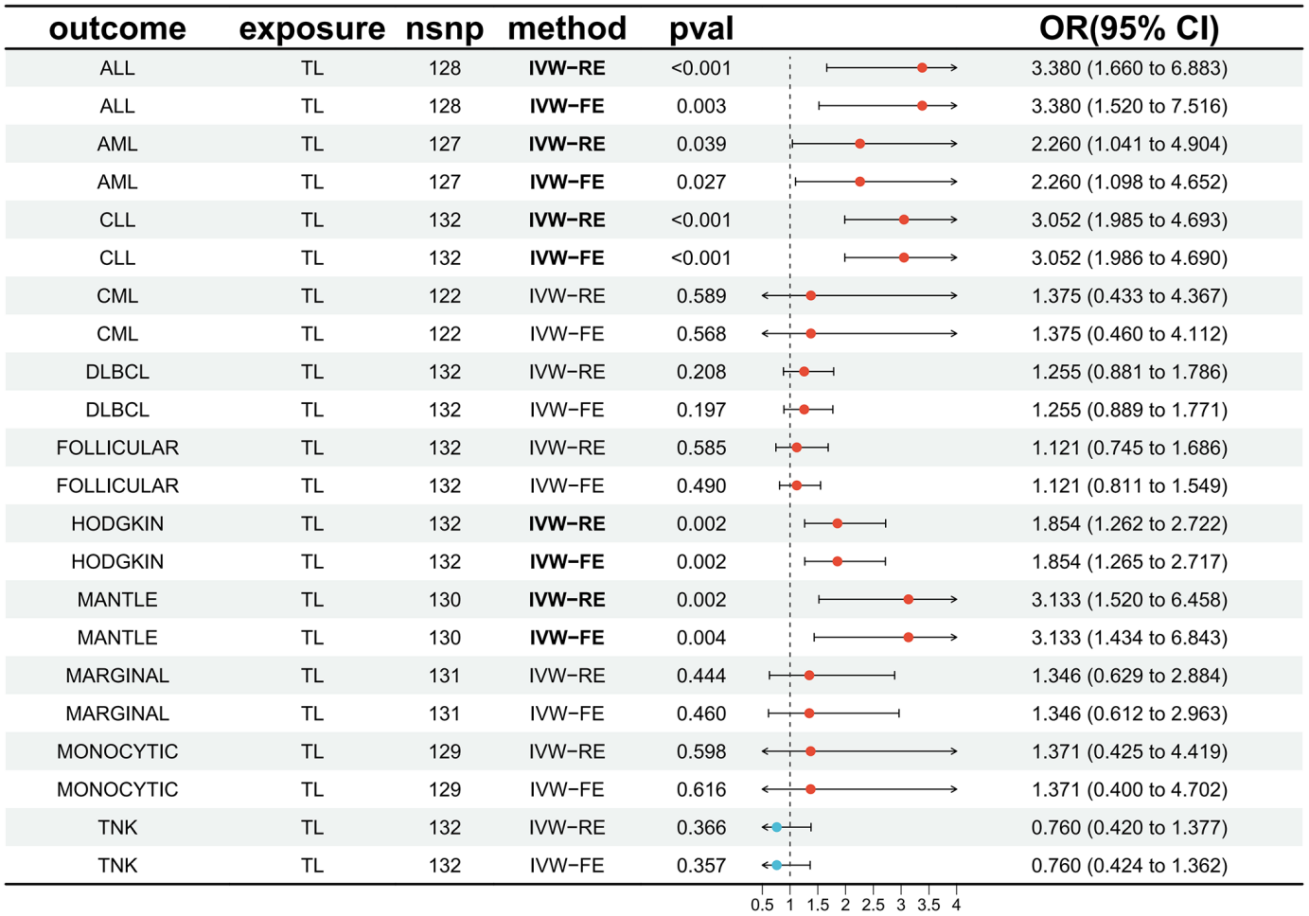
**Forest plot illustrating the association between genetically predicted TL and 11 HDs.** Abbreviations: TL: telomere length; HDs: hematologic diseases; IVW-RE: inverse variance weighted (random effects); IVW-FE: inverse variance weighted (fixed effects); OR: odds ratio; CI: confidence interval; ALL: acute lymphocytic leukemia; AML: acute myeloid leukemia; CLL: chronic lymphocytic leukemia; CML: chronic myeloid leukemia; DLBCL: diffuse large b-cell lymphoma; FOLLICULAR: follicular lymphoma; HODGKIN: Hodgkin lymphoma; MANTLE: mantle cell lymphoma; MARGINAL: marginal zone b-cell lymphoma; MONOCYTIC: monocytic leukemia; TNK: mature T/NK-cell lymphomas.

**Figure 3:**
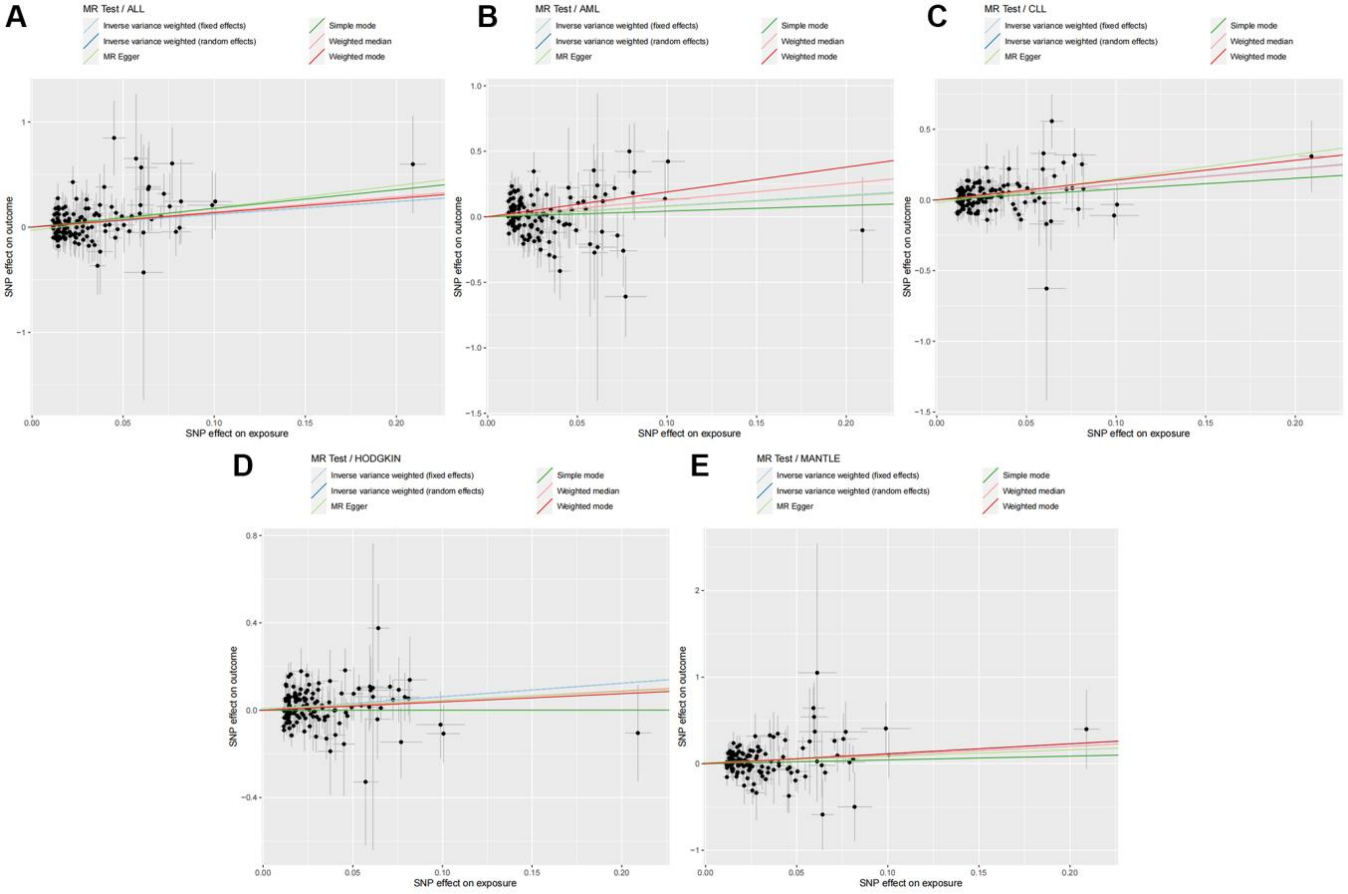
**Six MR methods demonstrated the causal effect of TL on HDs.** (**A**) Scatter plot illustrating the association between TL and ALL. (**B**) Scatter plot illustrating the association between TL and AML. (**C**) Scatter plot illustrating the association between TL and CLL. (**D**) Scatter plot illustrating the association between TL and HODGKIN. (**E**) Scatter plot illustrating the association between TL and MANTLE. Abbreviations: MR: Mendelian randomization; TL: telomere length; HDs: hematologic diseases; ALL: acute lymphocytic leukemia; AML: acute myeloid leukemia; CLL: chronic lymphocytic leukemia; HODGKIN: Hodgkin lymphoma; MANTLE: mantle cell lymphoma.

The impact of genetically predicted changes in TL on the risk of developing 11 HDs were analyzed, and the findings were presented in Figure 2. The IVW-FE/IVW-RE analysis revealed a significant causal relationship (*p* < 0.05) between genetically predicted TL changes and the risk of developing ALL (IVW-FE: OR = 3.380, 95% CI: 1.520–7.516, *p* = 0.003), AML (IVW-FE: OR = 2.260, 95% CI: 1.098–4.652, *p* = 0.027), CLL (IVW-FE: OR = 3.052, 95% CI: 1.986–4.690, *p* < 0.001), MANTLE (IVW-FE: OR = 3.133, 95% CI: 1.434–6.843, *p* = 0.004), and HODGKIN (IVW-FE: OR = 1.854, 95% CI: 1.265–2.717, *p* = 0.002). And among all the positive results, the results of the six MR methods were consistent (OR > 1), indicating that our analysis was reliable. The scatter plots also showed the consistency of the six MR methods and the reliability of the results (Figure 3).

The IVW-FE/IVW-RE results did not demonstrate a significant causal relationship (*p* > 0.05) between genetically predicted TL changes and the risk of developing CML (IVW-FE: OR = 1.375, 95% CI: 0.460–4.112, *p* = 0.568), DLBCL (IVW-FE: OR = 1.255, 95% CI: 0.889–1.771, *p* = 0.197), MARGINAL (IVW-FE: OR = 1.346, 95% CI: 0.612–2.963, *p* = 0.460), FOLLICULAR (IVW-RE: OR = 1.121, 95% CI: 0.745–1.686, *p* = 0.585), MONOCYTIC (IVW-FE: OR = 1.371, 95% CI: 0.400–4.702, *p* = 0.616), and TNK (IVW-FE: OR = 0.760, 95% CI: 0.424–1.362, *p* = 0.357). And among all the negative results, the results of other analytical methods also failed to establish a significant causal relationship (*p* > 0.05). The scatter plots for all negative results were available in the Supplementary File 2.

In the MR analysis, we assessed the heterogeneity and directional pleiotropy, as presented in Table 2. The results of the leave-one-out sensitivity analysis and the funnel plot results can be found in the Supplementary File 3. In the MR analysis using TL as the exposure, we observed a certain degree of heterogeneity only in the TL-FOLLICULAR MR analysis, hence selecting IVW-RE as the primary analytical result. No significant heterogeneity and directional pleiotropy were found in all other MR analyses, indicating a high level of reliability and reproducibility in the results. The results of all MR analyses were provided in Supplementary File 4.

**Table 2:** Pleiotropy and heterogeneity analyses.

**Outcome**	**Exposure**	**Heterogeneity**				**Pleiotropy**		
		**Q_mr egger**	**Q_pval_mr egger**	**Q_IVW**	**Q_pval_IVW**	**Egger intercept**	**SE**	***p***
ALL	TL	98.484	0.967	100.578	0.960	−0.031	0.021	0.150
AML	TL	145.068	0.106	145.072	0.118	0.001	0.020	0.957
CLL	TL	128.355	0.524	131.412	0.473	−0.020	0.011	0.083
CML	TL	134.178	0.178	134.678	0.187	0.021	0.031	0.505
DLBCL	TL	137.132	0.317	137.644	0.328	−0.006	0.009	0.487
FOLLICULAR	TL	207.599	<0.001	208.750	<0.001	0.009	0.011	0.397
HODGKIN	TL	131.681	0.442	132.293	0.452	0.008	0.010	0.438
MANTLE	TL	110.147	0.871	110.599	0.878	0.014	0.020	0.503
MARGINAL	TL	120.772	0.685	121.255	0.696	−0.014	0.021	0.488
MONOCYTIC	TL	115.320	0.763	115.445	0.779	−0.011	0.032	0.724
TNK	TL	134.146	0.384	136.102	0.362	−0.021	0.016	0.171

Abbreviations: IVW: inverse variance weighted; SE: standard error; ALL: acute lymphocytic leukemia; AML: acute myeloid leukemia; CLL: chronic lymphocytic leukemia; CML: chronic myeloid leukemia; DLBCL: diffuse large b-cell lymphoma; FOLLICULAR: follicular lymphoma; HODGKIN: Hodgkin lymphoma; MANTLE: mantle cell lymphoma; MARGINAL: marginal zone b-cell lymphoma; MONOCYTIC: monocytic leukemia; TNK: mature T/NK-cell lymphomas.

## DISCUSSION

In this study, we utilized genetic variations determining TL as a surrogate measure and employed MR analysis to explore the relationship between TL and the risk of 11 HDs. These findings merit our attention and necessitate a reevaluation of the potential diagnostic and therapeutic significance of telomeres in HDs. Additionally, it is noteworthy that these investigations analyzed the association between TL and the risk of HDs, which differs from studies analyzing the relationship between TL and the prognosis of HDs.

Acute lymphoblastic leukemia (ALL) is a malignant and invasive tumor that includes B-cell ALL (B-ALL) and T-cell ALL (T-ALL), caused by abnormal proliferation of lymphocytes in the bone marrow [19]. There have been numerous studies on the relationship between TL and the prognosis of ALL. In B-ALL patients, high telomerase activity, elevated telomere reverse transcriptase (TERT) expression, and telomere shortening were closely associated with poor prognosis [20, 21]. However, some studies have also found that telomere elongation was linked to poor prognosis in ALL [22]. Evidently, there was no consistent pattern in the function of telomeres and telomerase in ALL, especially considering the various potential atypical roles of TERT [23]. Our analysis revealed that genetically predicted longer TL increases the risk of ALL and suggests that longer TL is the primary driver of ALL onset; measuring the difference in TL length between tumor cells before the onset of ALL (before the malignant clone proliferates extensively) and healthy individuals may potentially predict the onset of ALL in advance. In terms of treatment, telomerase inhibitors (such as imetelstat) may enhance standard ALL therapy [23]; therefore, the prospect of combined targeted therapy against telomeres and telomerase in treating ALL is promising.

AML is a highly heterogeneous leukemia, influenced by multiple factors [24]. Recent research has focused on the relationship between TL and the prognosis of AML. Most studies have found that TL is shorter in AML patients compared to the general population [25, 26]. Shortened TL in AML patients was closely associated with prognosis and survival [27]. Additionally, some studies have observed that longer TL was linked to an increased risk of developing AML, which aligned with our analysis. Longer TL promoted cell proliferation and viability, thereby increasing the risk of AML [28]. This suggested that TL may serve as a potential biomarker for assessing the risk of developing AML. Several studies have also explored the possibility of targeting TL for therapeutic purposes. For instance, interfering with telomerase activity inhibited AML cell proliferation and viability [29]. Further elucidation of the regulatory mechanisms of TL and AML, as well as the development of therapeutic strategies targeting TL, could facilitate early prevention and diagnosis of AML, ultimately improving patient prognosis and survival rates.

CLL is a mature B-cell lymphoma, and multiple factors influence its development and prognosis. Several studies have shown that CLL patients tend to have shorter average TL compared to normal controls [30–32]. CLL with shorter TL often exhibited specific cytogenetic abnormalities and cell surface markers associated with poor prognosis and faster disease progression [33]. However, an earlier analysis indicated that genetic variants associated with longer TL were linked to an increased risk of developing CLL [34]. This suggests that a baseline propensity for longer telomeres may provide opportunities for premalignant cells to undergo malignant transformation, while subsequent telomere depletion leads to TL shortening [35]. Although the sample size and the study design may influence the results, they were consistent with our analysis using the latest data. Therefore, TL may play a crucial role in the occurrence, development, and prognosis of CLL. The influence of telomeres on the pathogenesis of CLL was complex, underscoring the importance of unraveling the biological characteristics of telomeres at different stages of CLL.

HODGKIN is a malignant tumor of the lymphoid system, and its pathogenesis is not fully understood. Telomeres may play a role in the occurrence and development of HODGKIN. Studies have found that patients with HODGKIN may have shorter TL at diagnosis compared to healthy individuals [36]. HODGKIN also exhibited telomere dysfunction, activation of telomerase, and alternative lengthening of telomeres (ALT) [37, 38]. Telomere dysfunction in HODGKIN includes extremely short telomeres, altered telomere numbers, telomere aggregation, and changes in 3D spatial structure [39]. Furthermore, previous studies have shown that sequential inhibition of telomerase and ALT promotes the cell death in HODGKIN [40]. Our findings indicated that genetic variants associated with longer TL were associated with an increased risk of HODGKIN. In fact, several scholars have suggested that both shorter and longer telomeres may contribute to carcinogenesis, and that an optimal TL represented a balance between cell proliferation, senescence, and control [41–43]. Cells with longer TL had greater proliferative and mutational potential, leading to the onset of HODGKIN, while shortened TL was often correlated with disease severity after onset [44, 45].

MANTLE lymphoma is a non-Hodgkin lymphoma (NHL) originating from mature B cells, characterized by specific immunophenotype and recurrent genetic abnormalities [46]. Studies have shown that TERT promoter (TERTp) mutations leading to higher TERT expression levels were associated with longer telomeres in MANTLE, particularly in homozygous mutants; TERTp mutations may contribute to a more aggressive clinical behavior of MANTLE and could be associated with poorer prognosis [47]. A study on TL in 73 MANTLE and 20 normal B cell samples found highly variable TL in MANTLE (range, 2.2-13.8 kb; median, 4.3 kb) and significant telomeres dysfunction, but observed no association with any biological or clinical features [48]. Currently, research on telomere biology in MANTLE is limited, and our analysis predictive of genetic variations in TL has revealed a relationship with the risk of MANTLE onset, necessitating large-scale cohort studies to validate these findings.

An increasing body of evidence suggests that longer telomeres may be associated with an increased risk of NHL. A prospective study involving 107 male NHL cases and 107 matched controls found that longer relative TL may be linked to an increased risk of NHL [43]. Nested case-control studies conducted on 464 lymphoma cases and 464 matched controls from the EPIC cohort also revealed an association between longer TL and an increased risk of B-cell lymphoma, particularly in DLBCL and FOLLICULAR subtypes [49]. TL in B lymphocytes infected with Epstein-Barr virus gradually increased and was associated with the accumulation of early granulocyte leukemia bodies [50]. Another study demonstrated a positive correlation between genetically predicted longer TL and the risk of four NHL subtypes (CLL/SLL, DLBCL, FOLLICULAR, and MANTLE) [51]. Currently, there is a lack of research on the relationship between TL and the risk of CML, MONOCYTIC, and TNK. However, in CML patients, telomeres were often shortened [5]. In CML (BCR/ABL-positive), accelerated telomere shortening has been linked to the disease progression, risk score, and treatment response [52]. Even though our analysis has not found an association between genetically predicted TL and the risk of CML, DLBCL, FOLLICULAR, MARGINAL, MONOCYTIC, and TNK, when combined with previous research, there still seems to be an association between TL and the risk of certain diseases.

Despite the effective and stable results of our analysis, there were some limitations. Firstly, the sample size of the outcome factors was small, and the statistical efficacy could be further improved by conducting future studies with a larger sample size. Secondly, due to the limitation in sample size, our MR analysis failed to stratify specific factors such as age and sex, and only obtained summary-level statistics. Lastly, the individuals included in our analysis were of European populations, so caution should be exercised when generalizing these findings to other populations.

## CONCLUSIONS

In conclusion, our findings indicate that a longer genetically predicted TL is associated with an increased risk of developing ALL, AML, CLL, MANTLE, and HODGKIN. However, there is no significant causal relationship between genetically predicted TL changes and the risk of developing CML, DLBCL, FOLLICULAR, MARGINAL, MONOCYTIC, and TNK. This study further supports the notion that cells with longer TL have greater proliferative and mutational potential, leading to an increased risk of certain HDs. Future research should focus on exploring the early diagnosis and therapeutic value of telomeres in patients with HDs.

## Supplementary Materials

Supplementary File 1

Supplementary File 2

Supplementary File 3

Supplementary File 4
