# Supplementary material for: Genetically predicted telomere length and the risk of 11 hematological diseases: a Mendelian randomization study
Source: Aging (Albany NY). 2024 Feb 22;16(5):4270–81. doi: 10.18632/aging.205583 (PMC10968687; doi:10.18632/aging.205583)
Supplement: Supplementary File 2 [file aging-16-205583-s002.pdf]

Supplementary File 2. The scatter plots for all negative results.

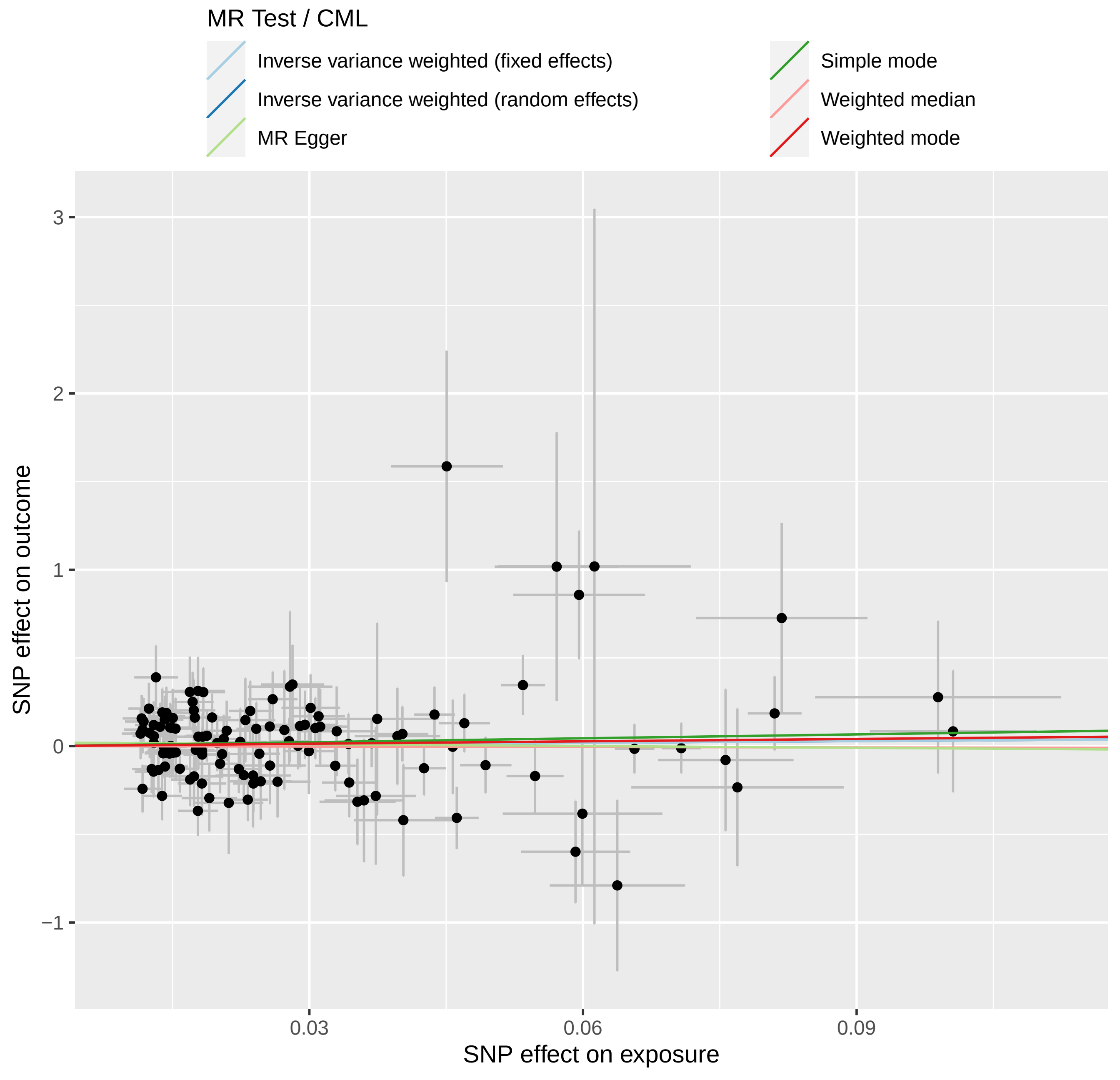

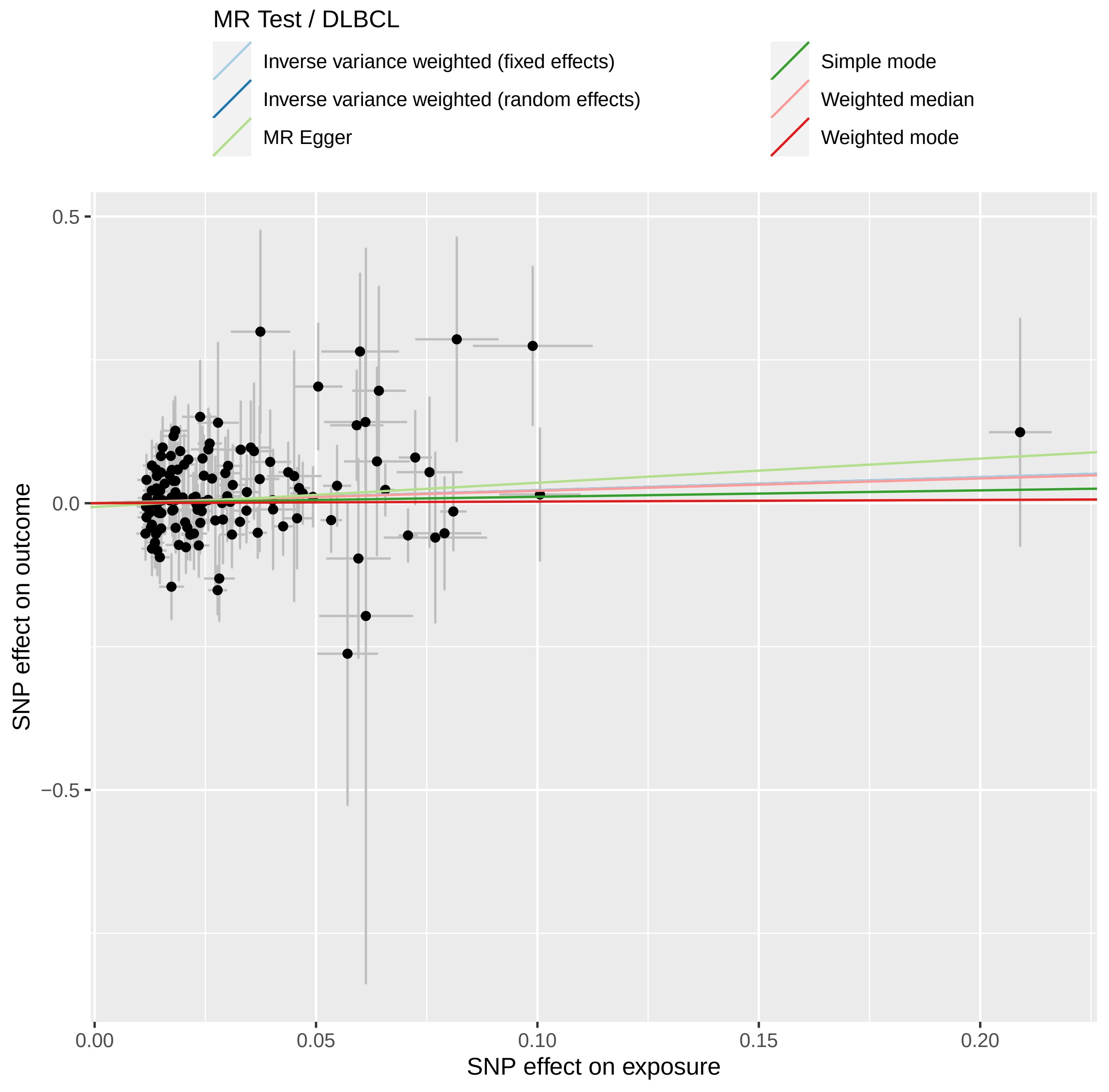

MR Test / DLBCL
Inverse variance weighted (fixed effects)
Inverse variance weighted (random effects)
MR Egger
Simple mode
Weighted median
Weighted mode
0.5
0.0
−0.5
0.00
0.05
0.10
0.15
0.20
SNP effect on outcome
SNP effect on exposure

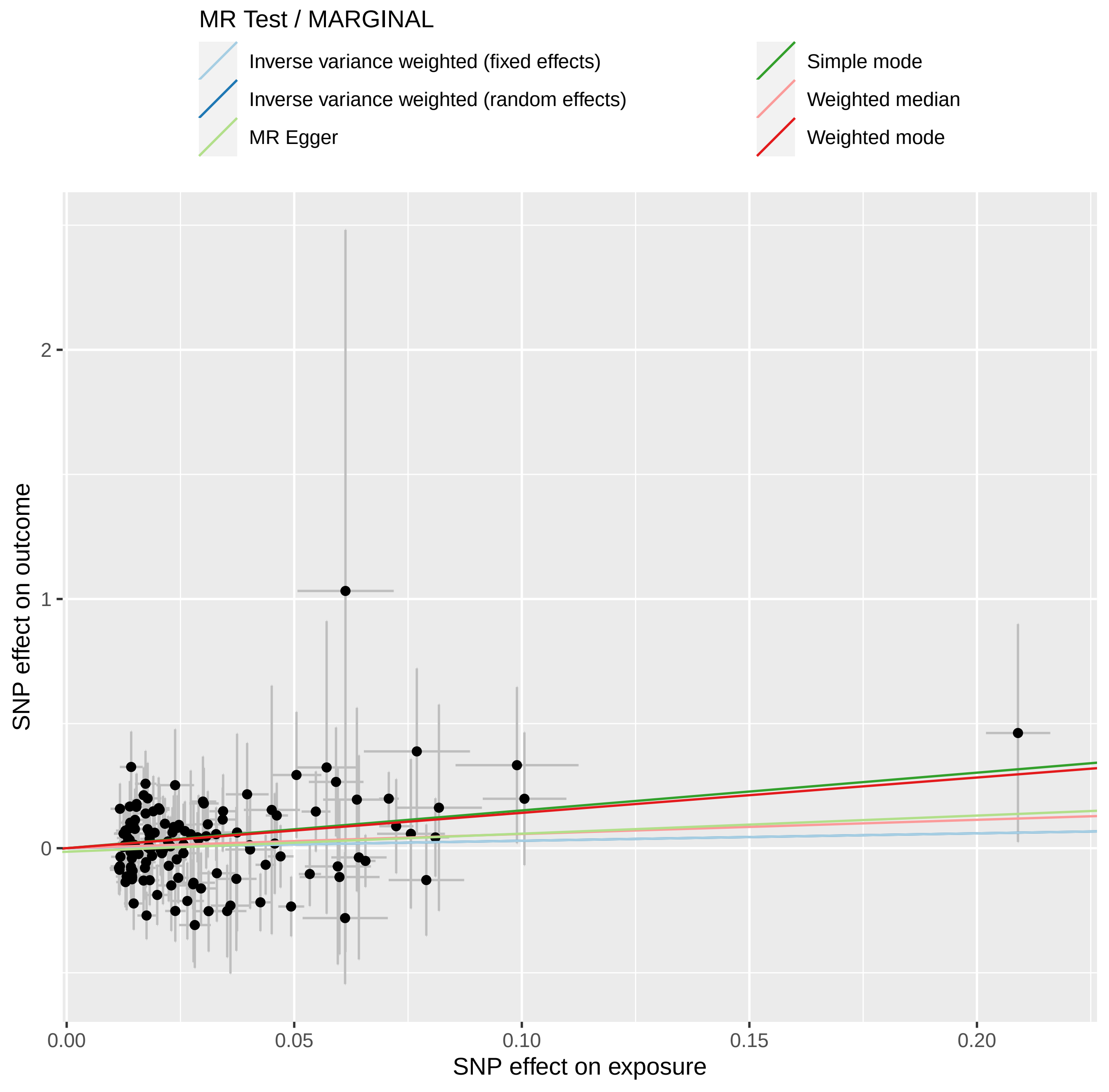

MR Test / MARGINAL
Inverse variance weighted (fixed effects)
Inverse variance weighted (random effects)
MR Egger
Simple mode
Weighted median
Weighted mode
SNP effect on outcome
SNP effect on exposure
0
1
2
0.00
0.05
0.10
0.15
0.20

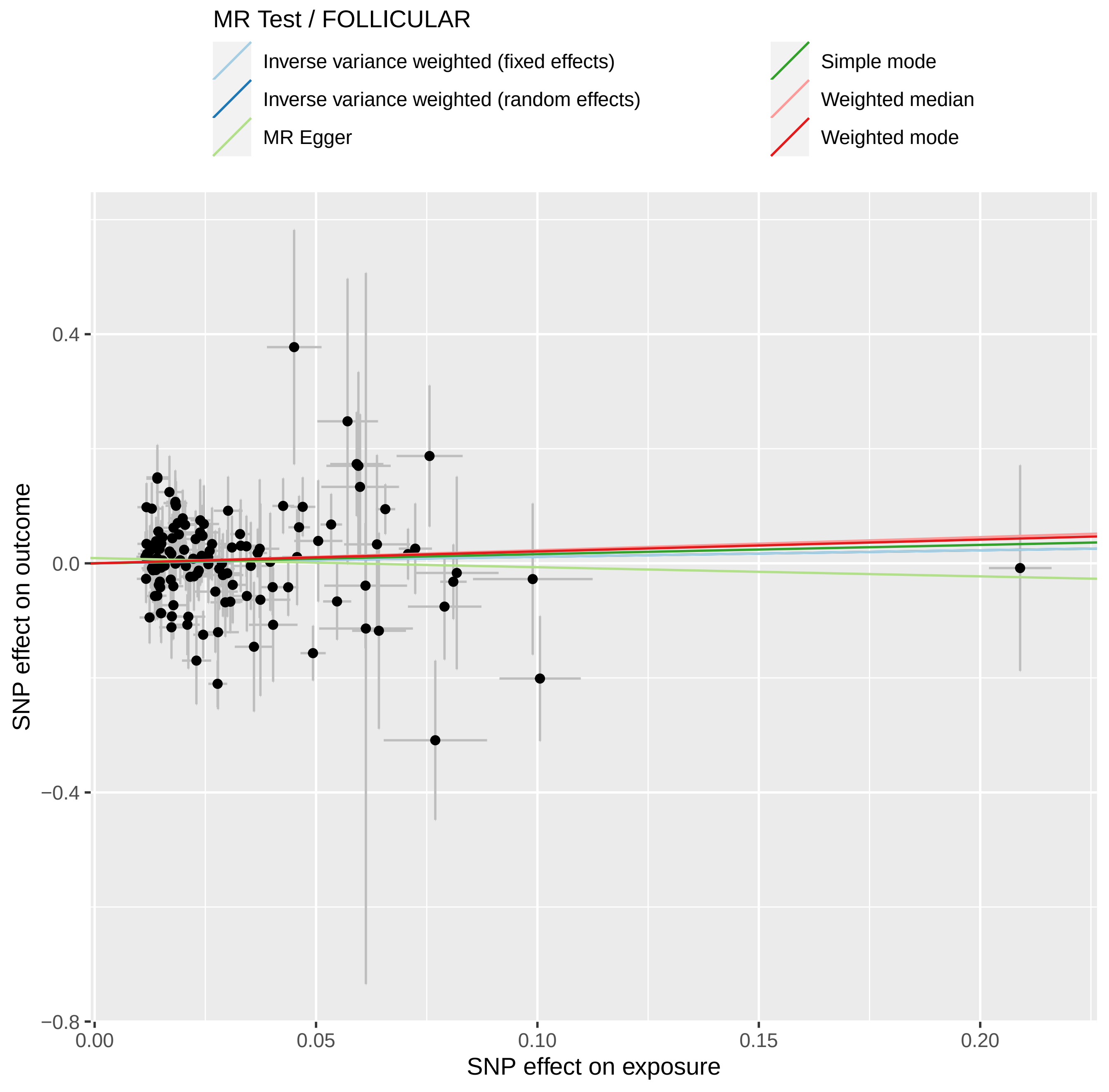
MR Test / FOLLICULAR
Inverse variance weighted (fixed effects)
Inverse variance weighted (random effects)
MR Egger
Simple mode
Weighted median
Weighted mode
SNP effect on outcome
0.4
0.0
−0.4
−0.8
0.00
0.05
0.10
0.15
0.20
SNP effect on exposure

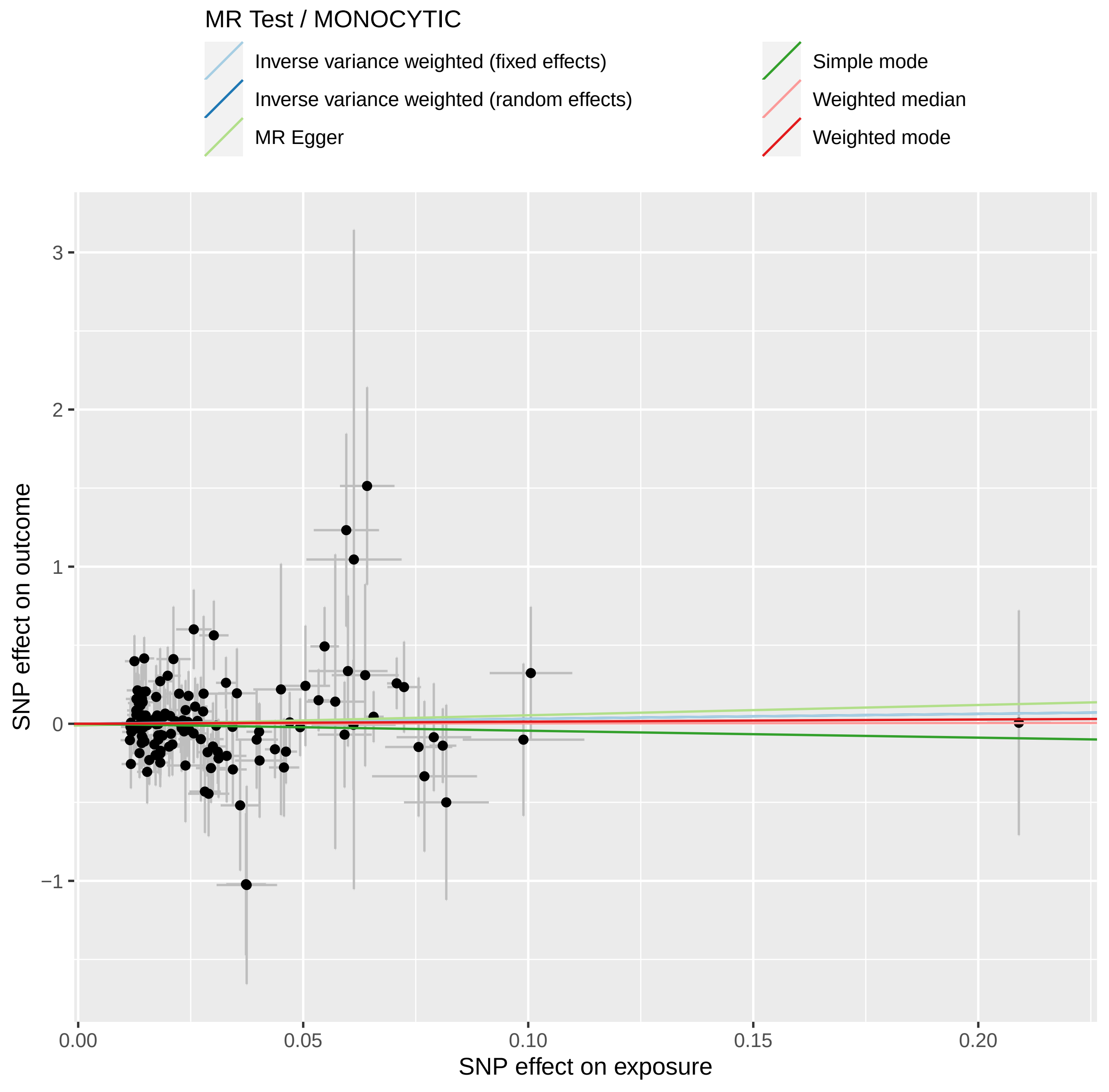
MR Test / MONOCYTIC
Inverse variance weighted (fixed effects)
Inverse variance weighted (random effects)
MR Egger
Simple mode
Weighted median
Weighted mode
SNP effect on outcome
3
2
1
0
−1
0.00
0.05
0.10
0.15
0.20
SNP effect on exposure

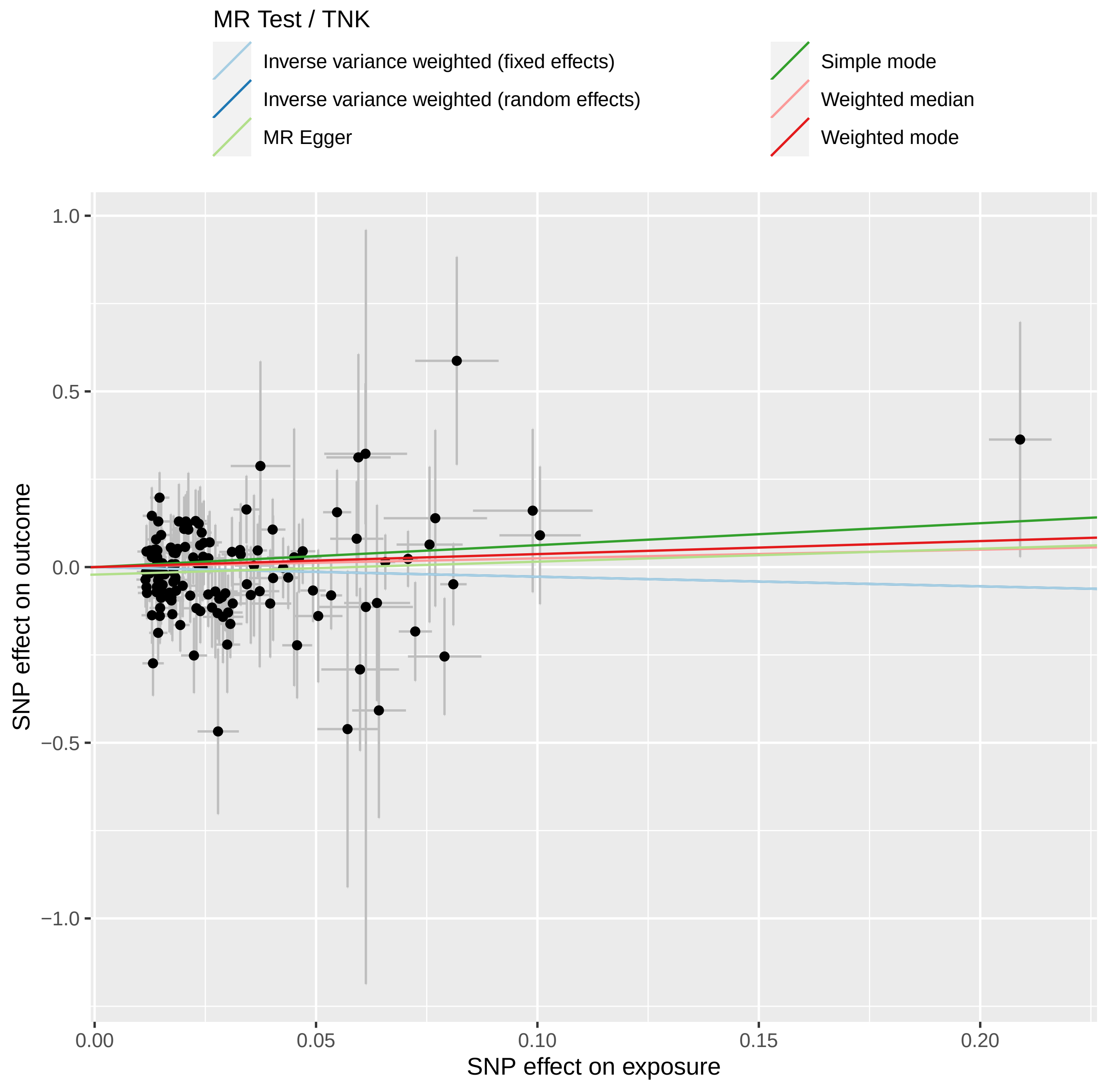

MR Test / TNK
Inverse variance weighted (fixed effects)
Inverse variance weighted (random effects)
MR Egger
Simple mode
Weighted median
Weighted mode
SNP effect on outcome
SNP effect on exposure
1.0
0.5
0.0
−0.5
−1.0
0.00
0.05
0.10
0.15
0.20
