# Supplementary material for: Genetically predicted telomere length and the risk of 11 hematological diseases: a Mendelian randomization study
Source: Aging (Albany NY). 2024 Feb 22;16(5):4270–81. doi: 10.18632/aging.205583 (PMC10968687; doi:10.18632/aging.205583)
Supplement: Supplementary File 3 [file aging-16-205583-s003.pdf]

Supplementary File 3. The leave-one-out sensitivity analysis plots and the funnel plots.

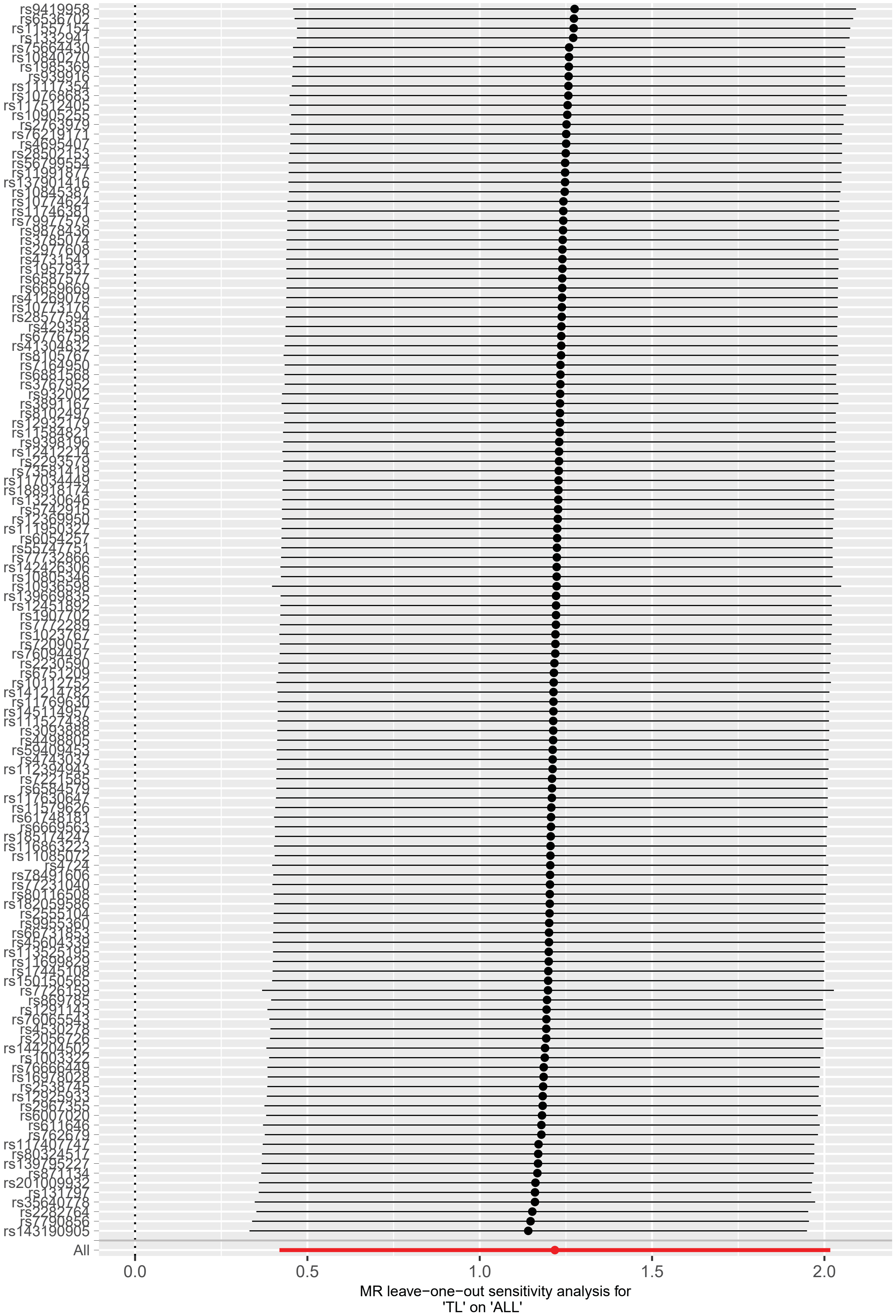

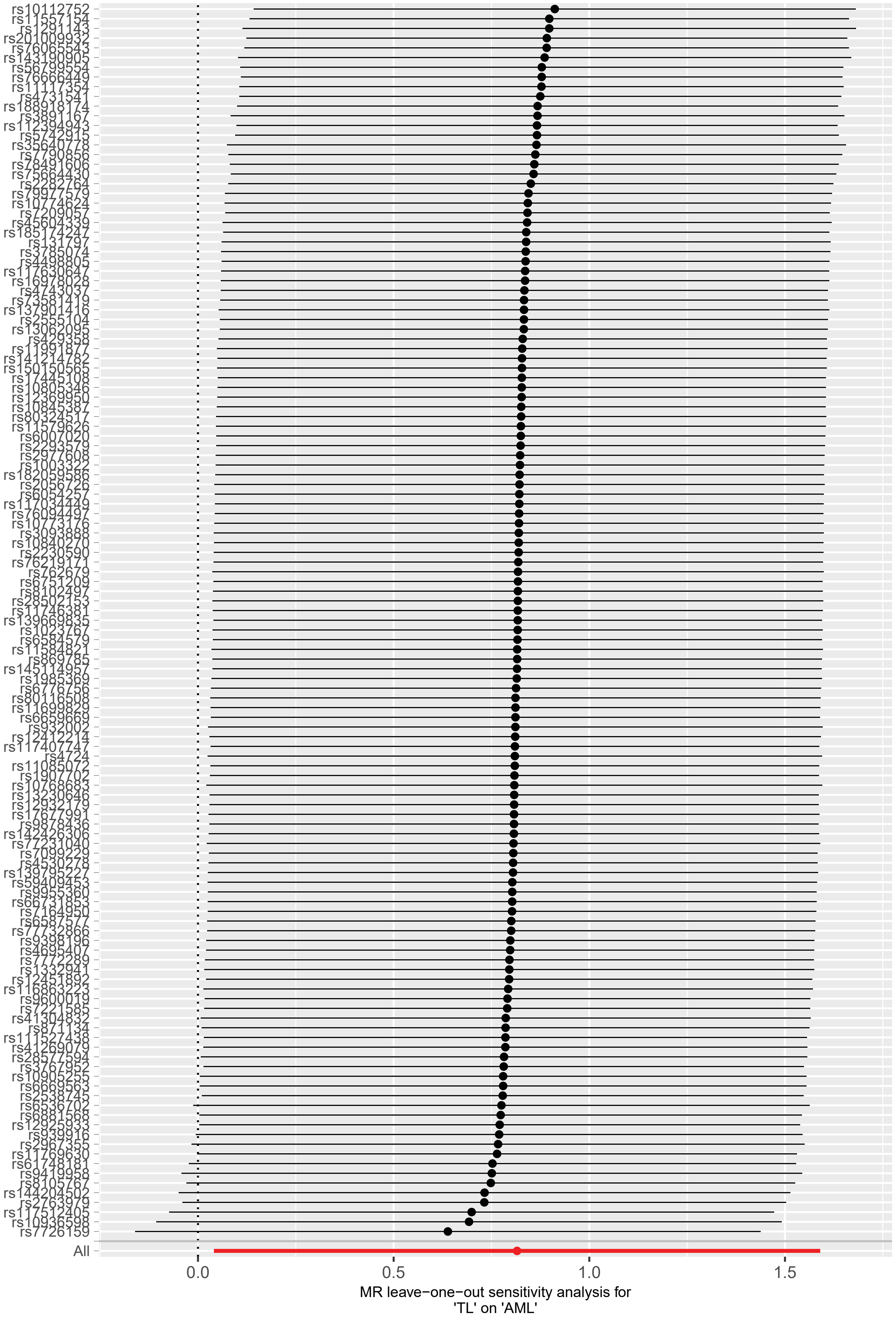
rs10112752
rs7726159
All
0.0
0.5
1.0
1.5
MR leave−one−out sensitivity analysis for
'TL' on 'AML'

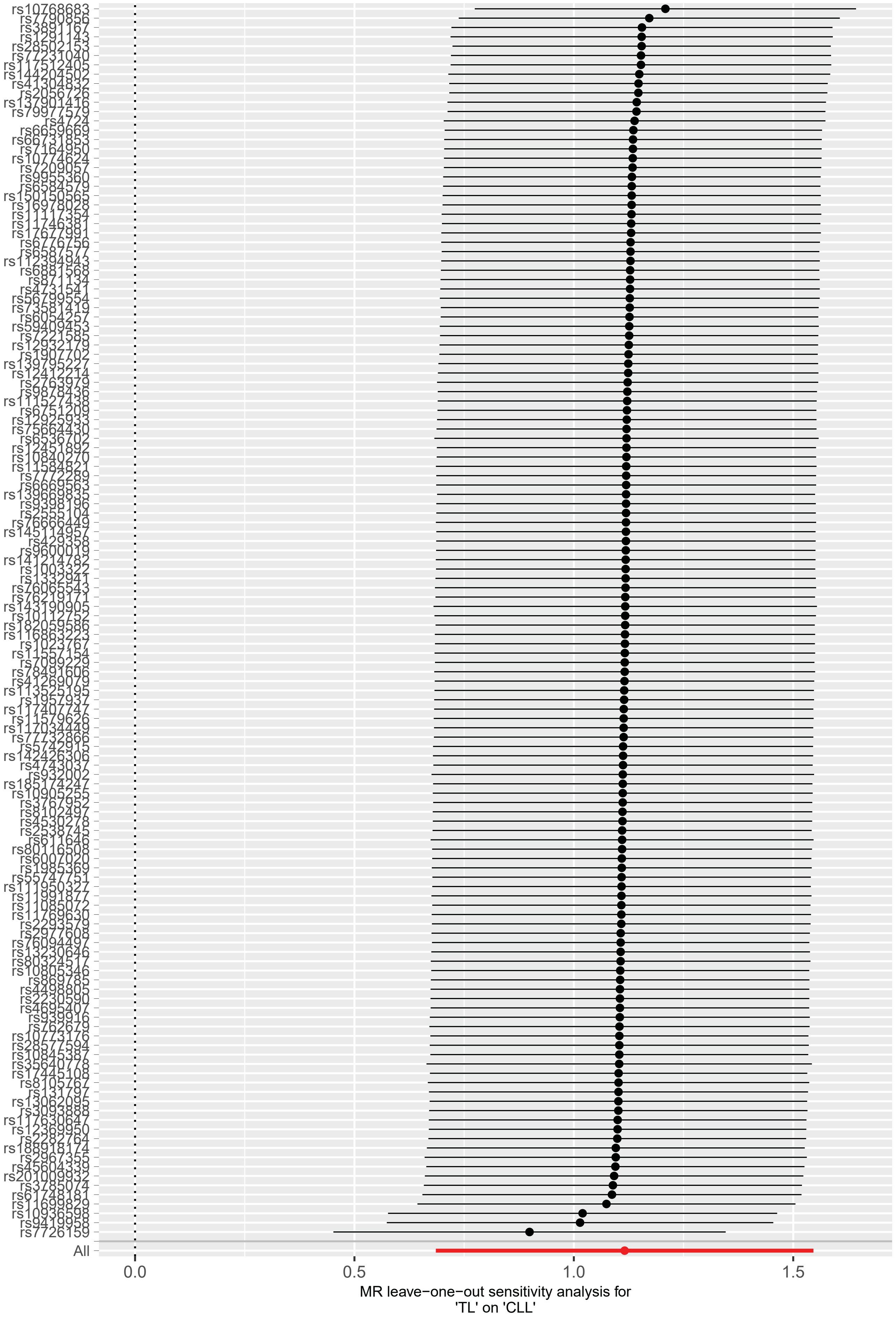

rs10768683
rs7790856
All
0.0
0.5
1.0
1.5
MR leave−one−out sensitivity analysis for
'TL' on 'CLL'

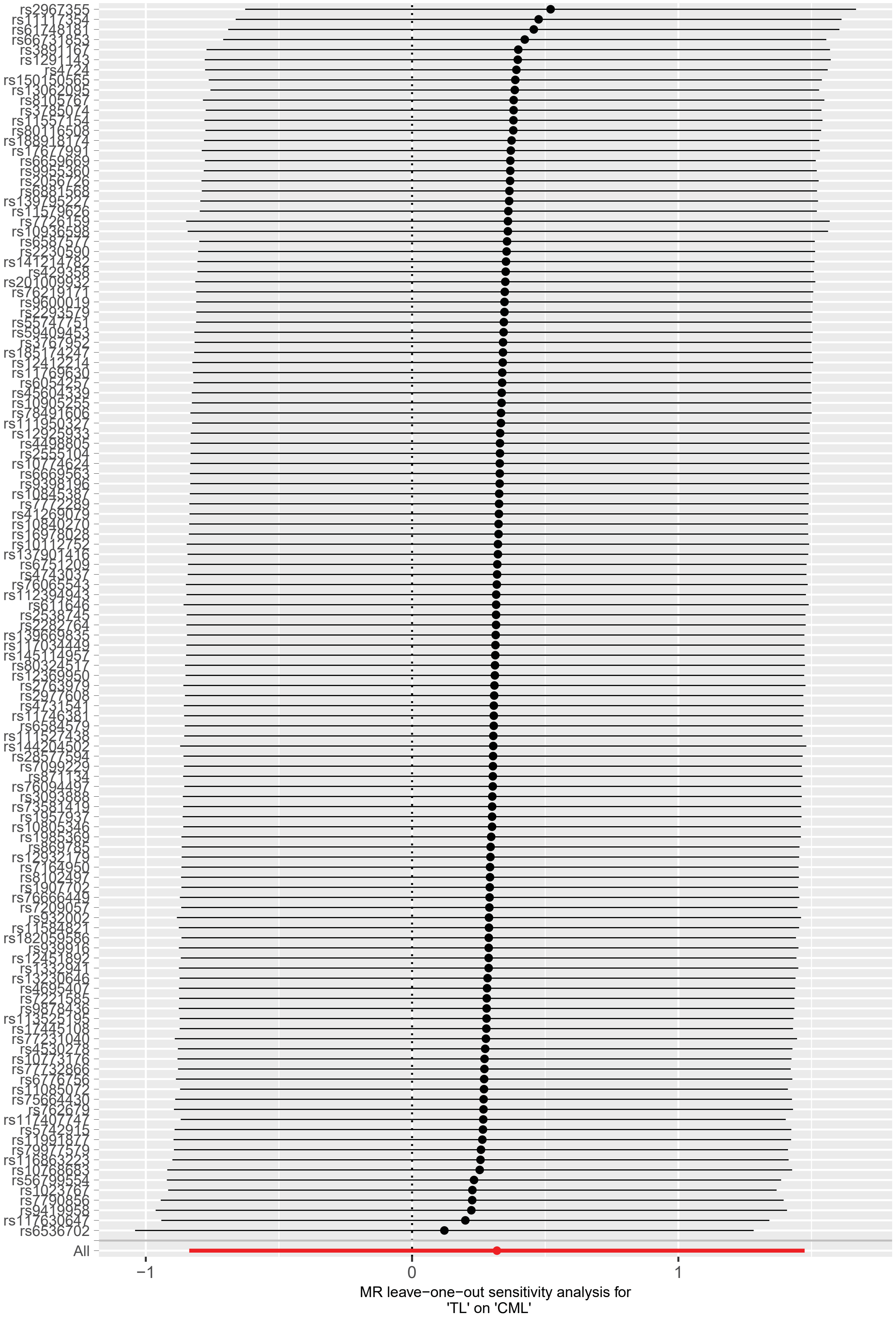
rs2967355
All
−1
0
1
MR leave−one−out sensitivity analysis for
'TL' on 'CML'

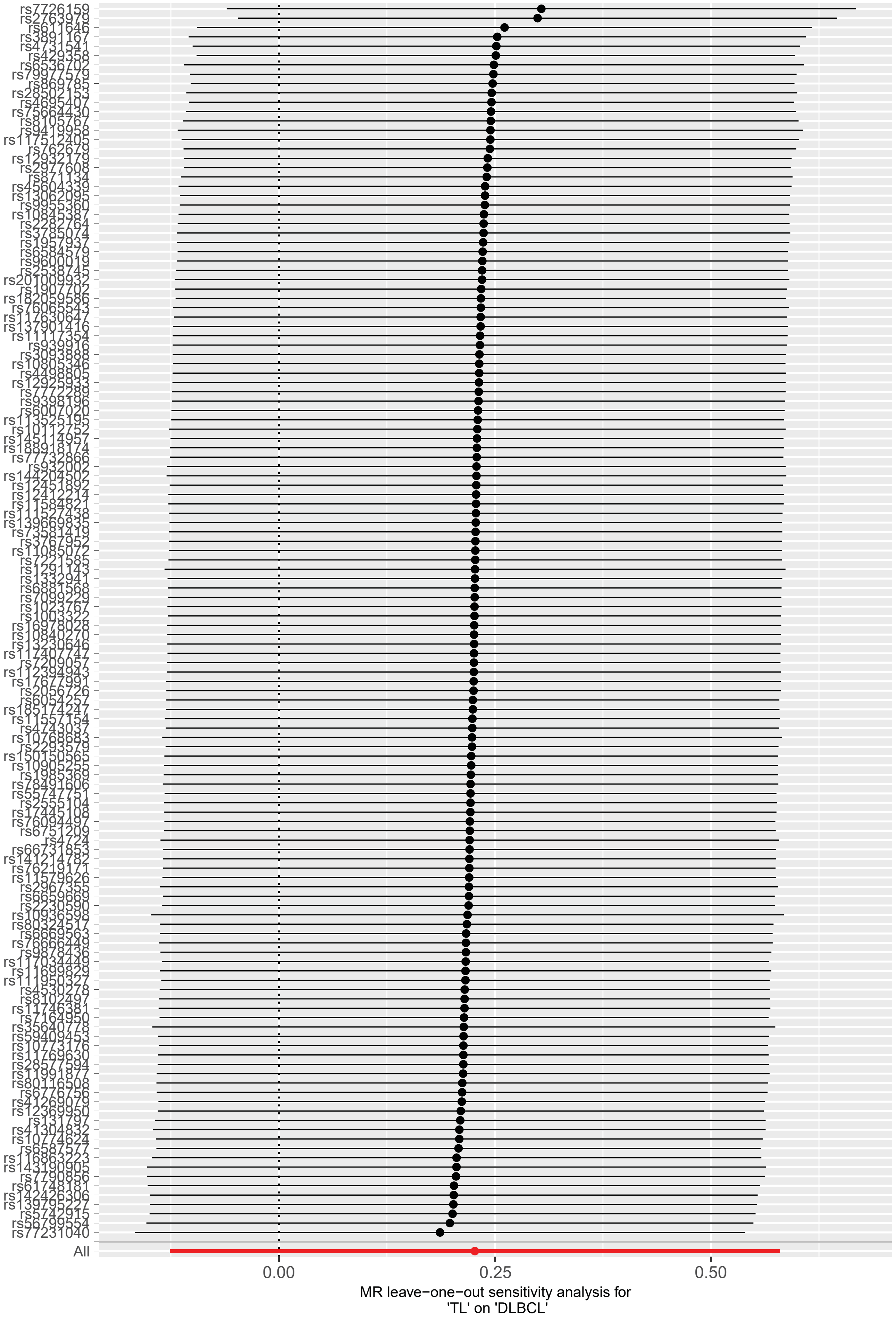
All
0.00
0.25
0.50
MR leave−one−out sensitivity analysis for
'TL' on 'DLBCL'

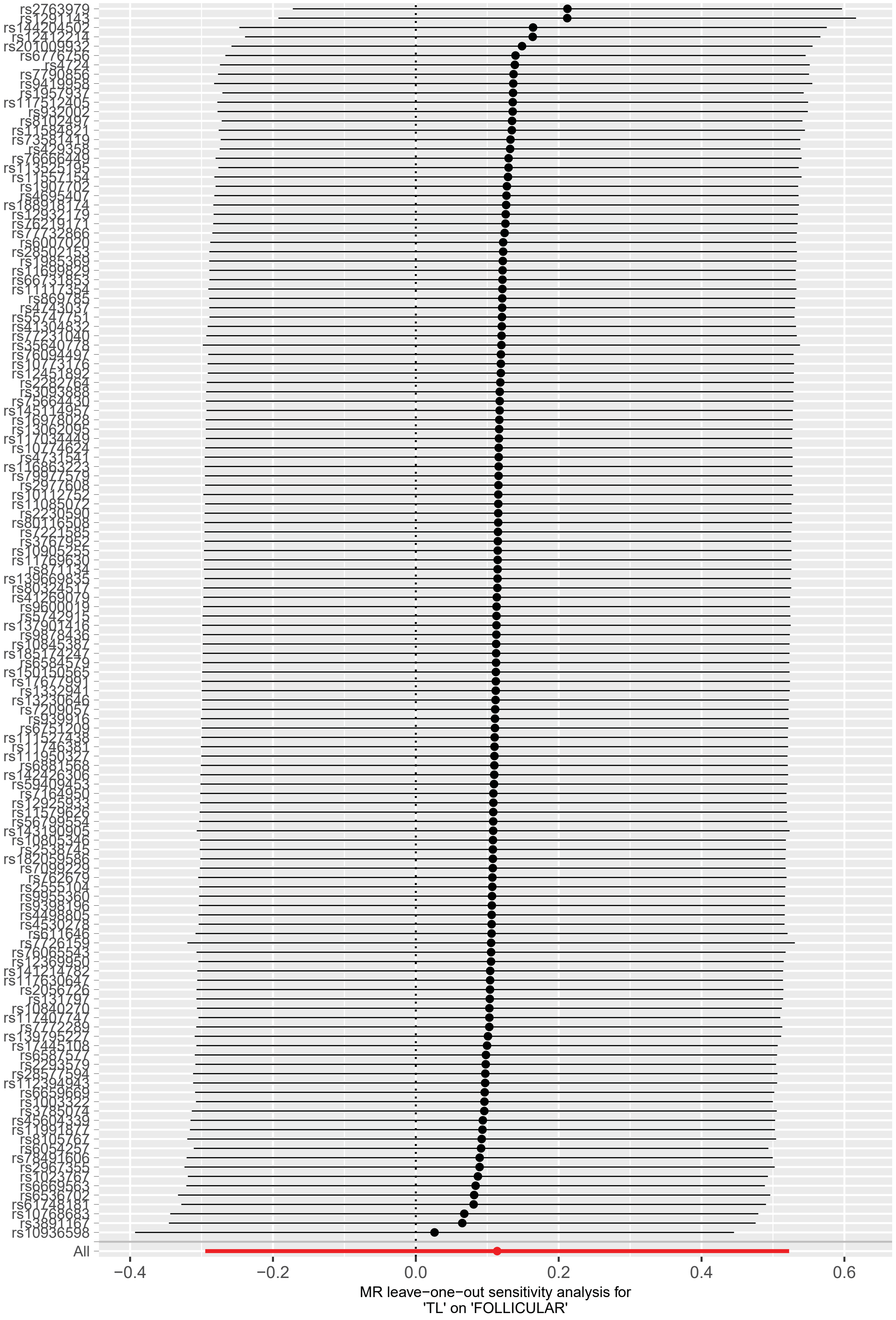
All
−0.4
−0.2
0.0
0.2
0.4
0.6
MR leave−one−out sensitivity analysis for
'TL' on 'FOLLICULAR'

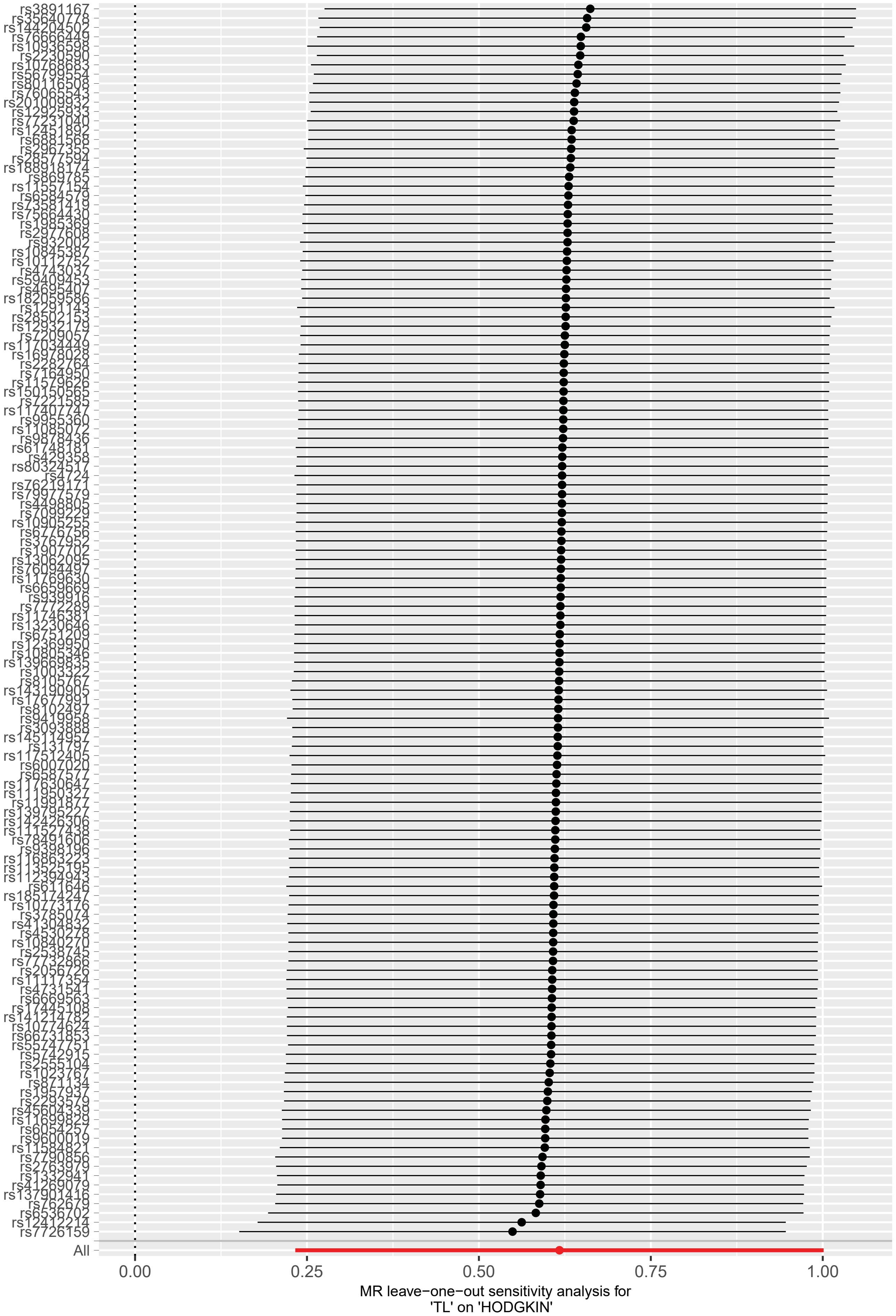
All
0.00
0.25
0.50
0.75
1.00
MR leave−one−out sensitivity analysis for
'TL' on 'HODGKIN'

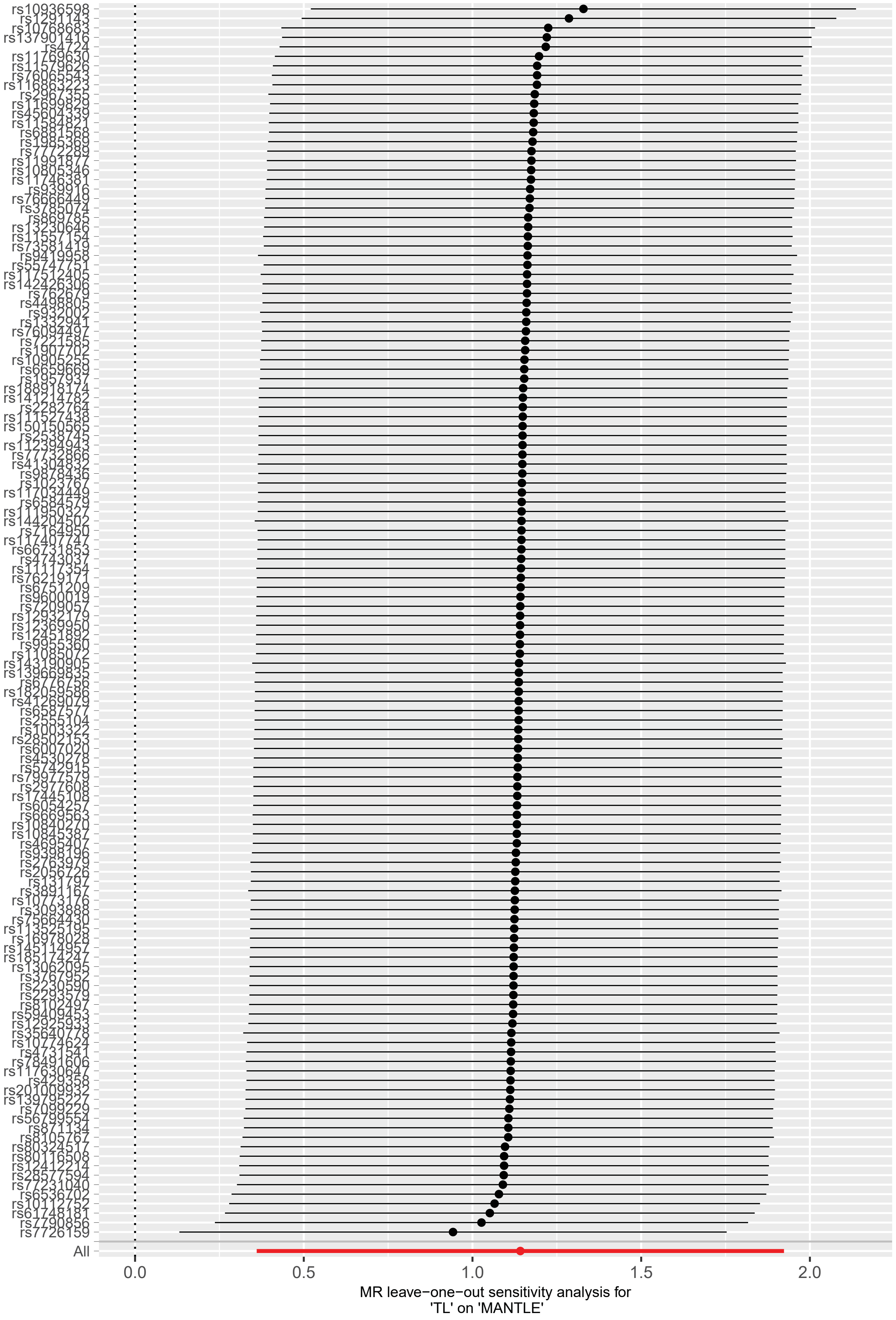
rs10936598
rs7726159
All
0.0
0.5
1.0
1.5
2.0
MR leave−one−out sensitivity analysis for
'TL' on 'MANTLE'

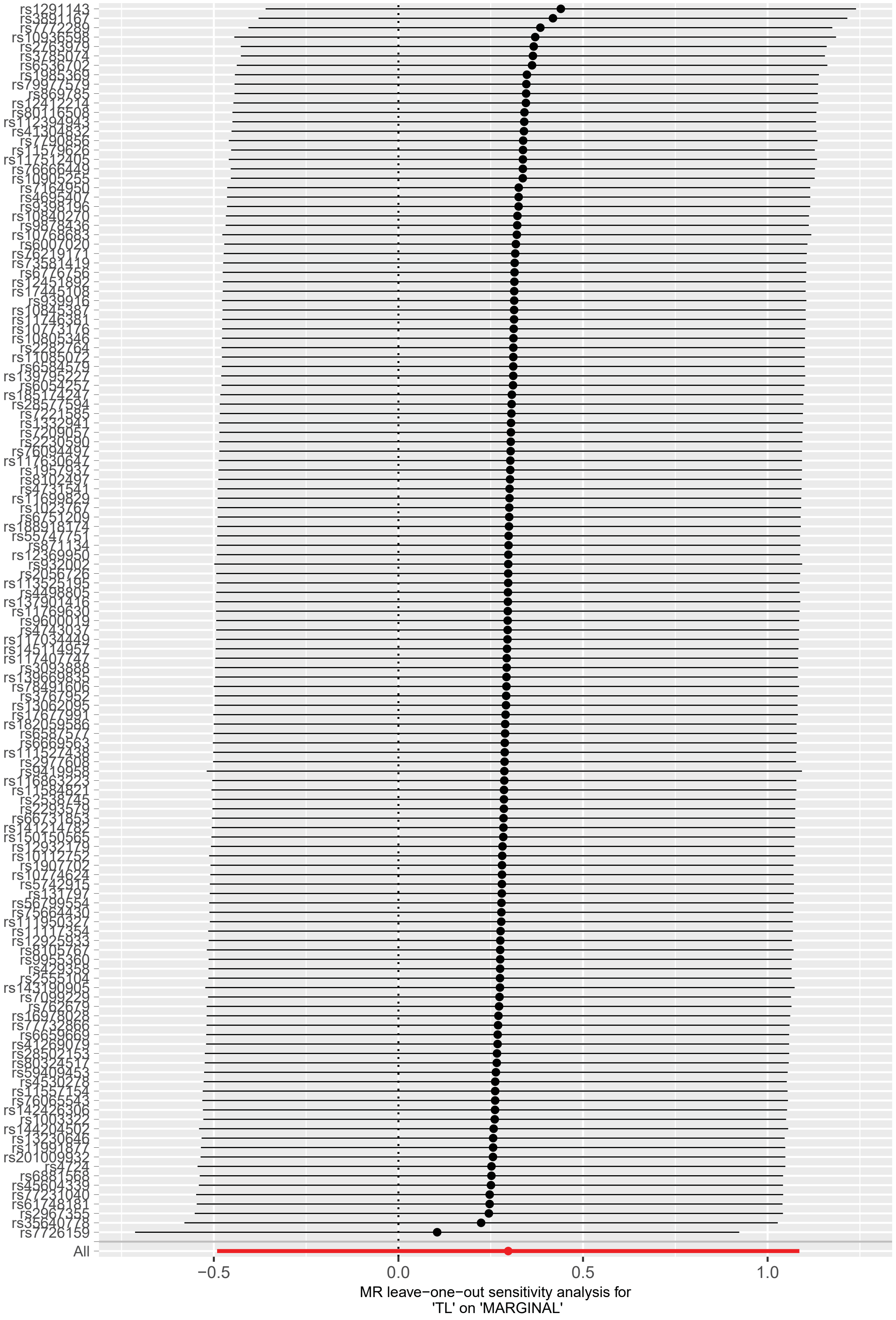

rs1291143
rs726159
All
−0.5
0.0
0.5
1.0
MR leave−one−out sensitivity analysis for
'TL' on 'MARGINAL'

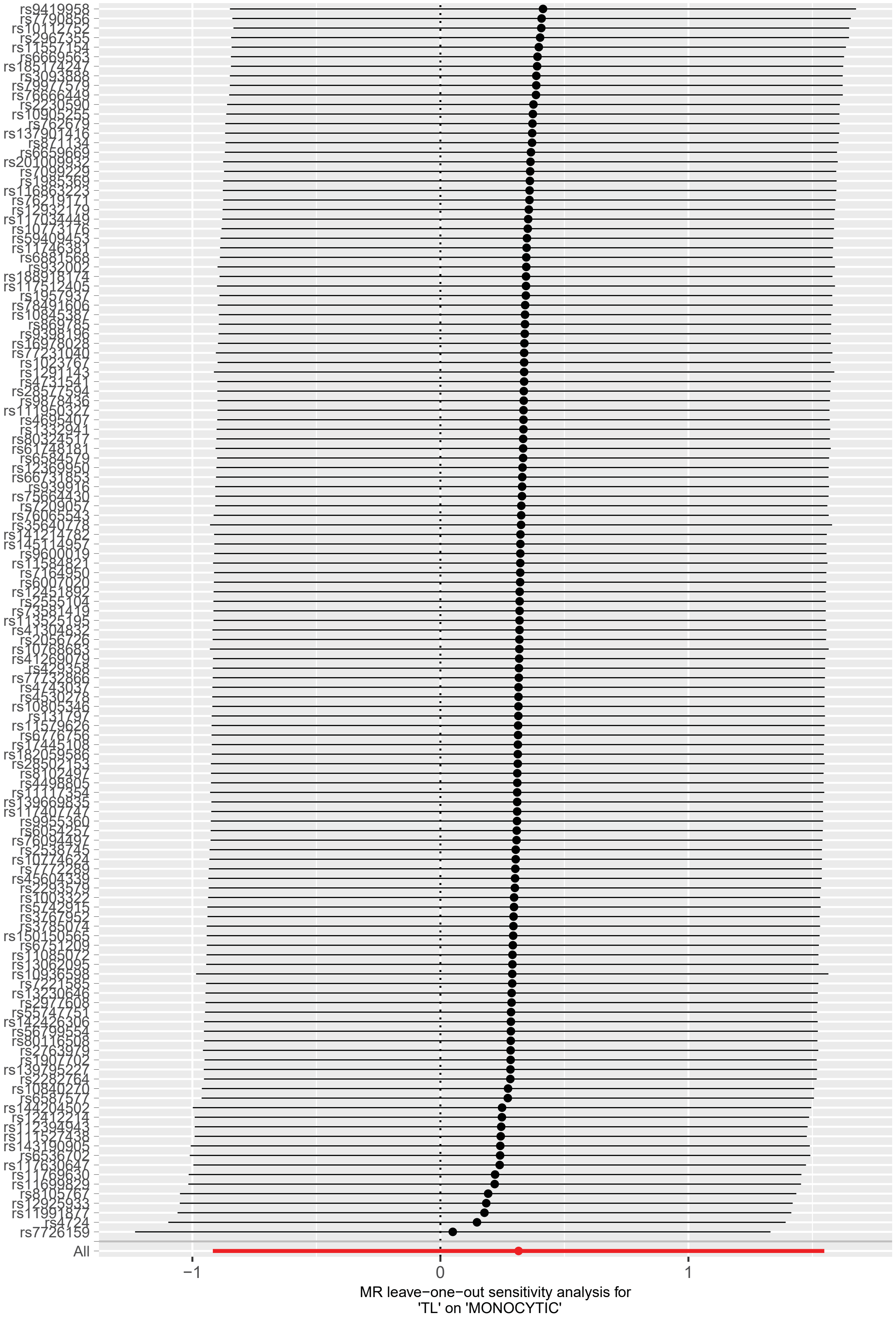
rs9419958
rs7726159
All
−1
0
1
MR leave−one−out sensitivity analysis for
'TL' on 'MONOCYTIC'

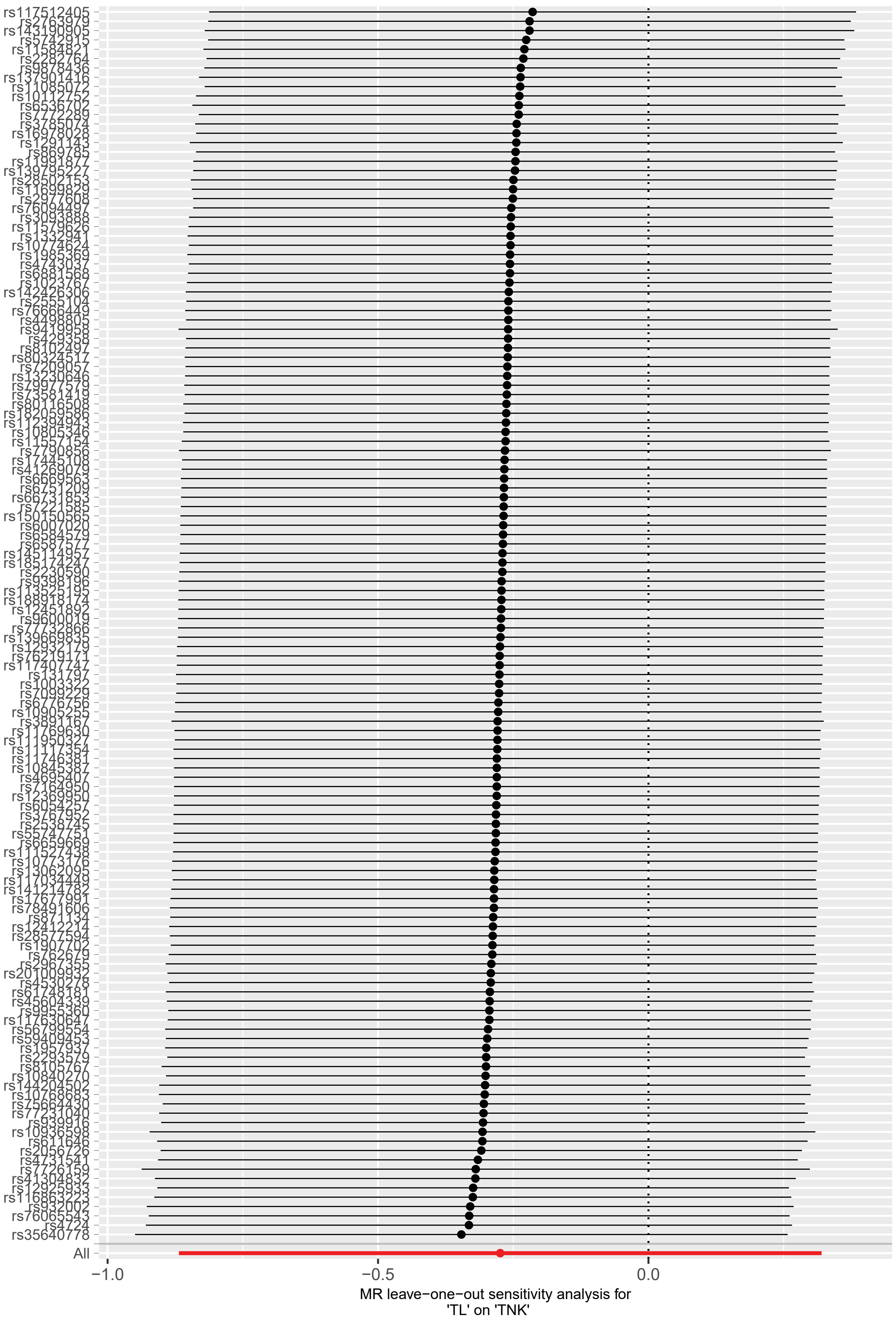

rs117512405
rs35640778
All
−1.0
−0.5
0.0
MR leave−one−out sensitivity analysis for
'TL' on 'TNK'

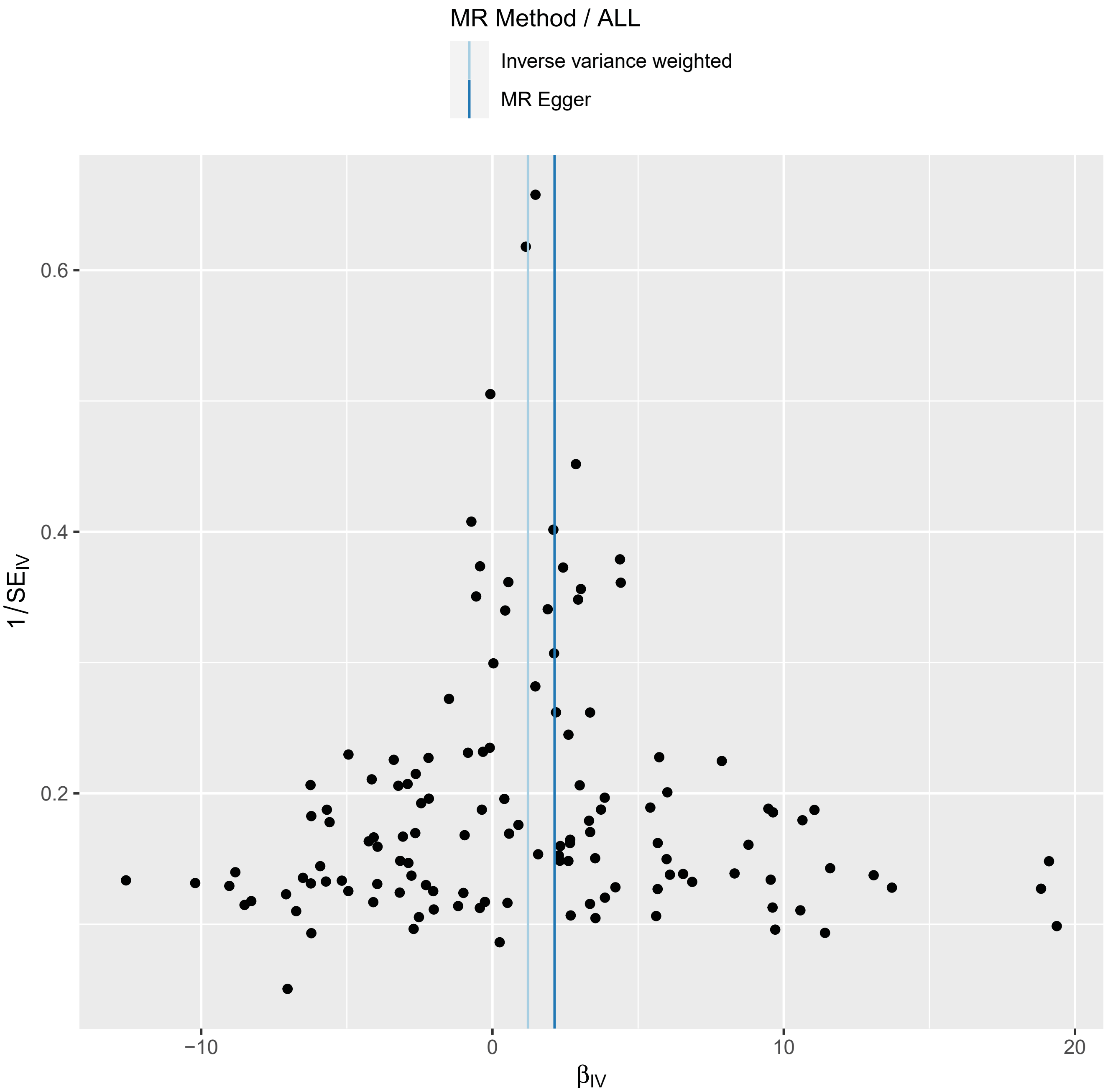
MR Method / ALL
Inverse variance weighted
MR Egger
0.6
0.4
0.2
1/SE$_{IV}$
−10
0
10
20
β$_{IV}$

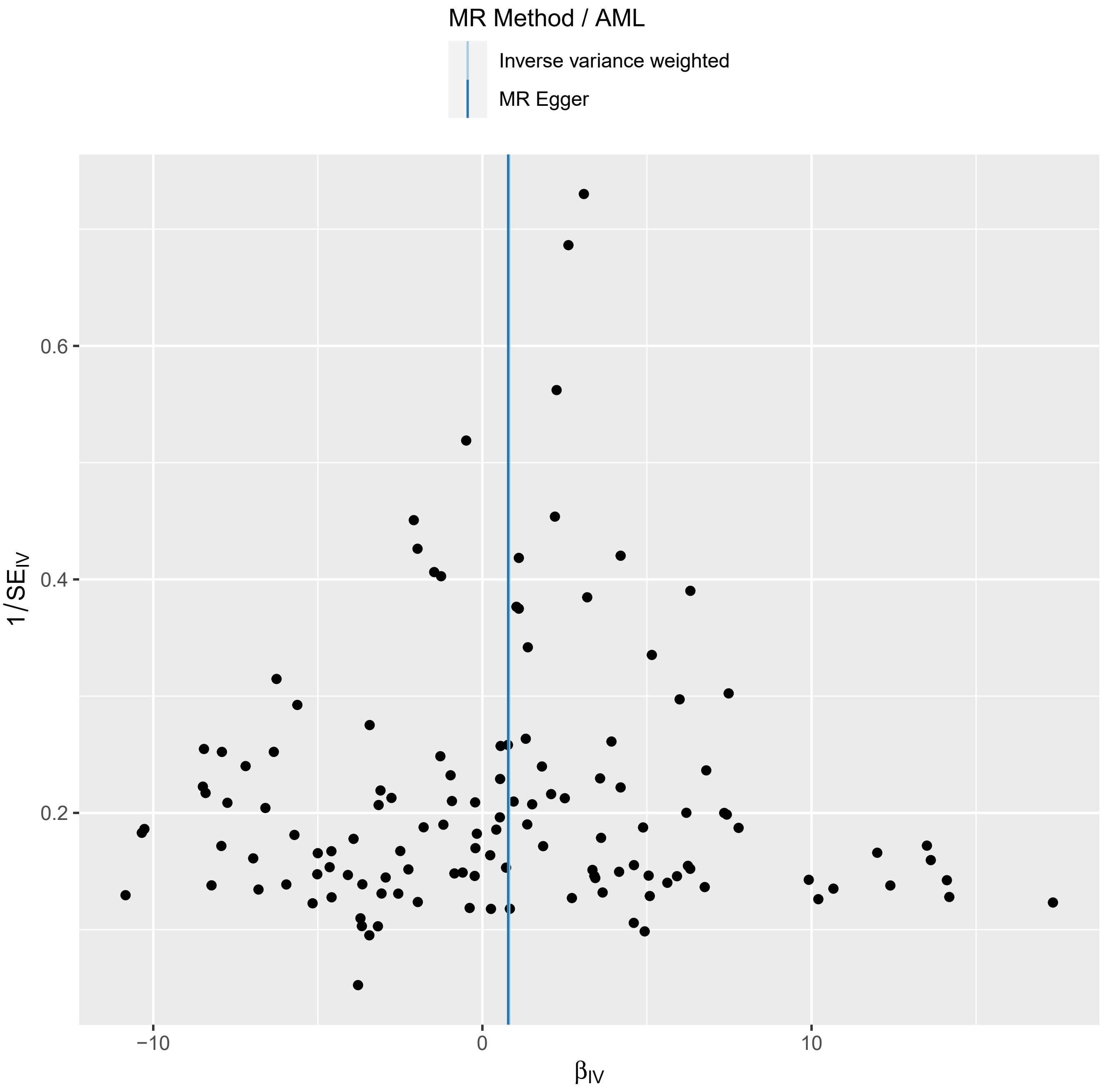
MR Method / AML
Inverse variance weighted
MR Egger
0.6
0.4
0.2
1/SE$_{IV}$
−10
0
10
β$_{IV}$

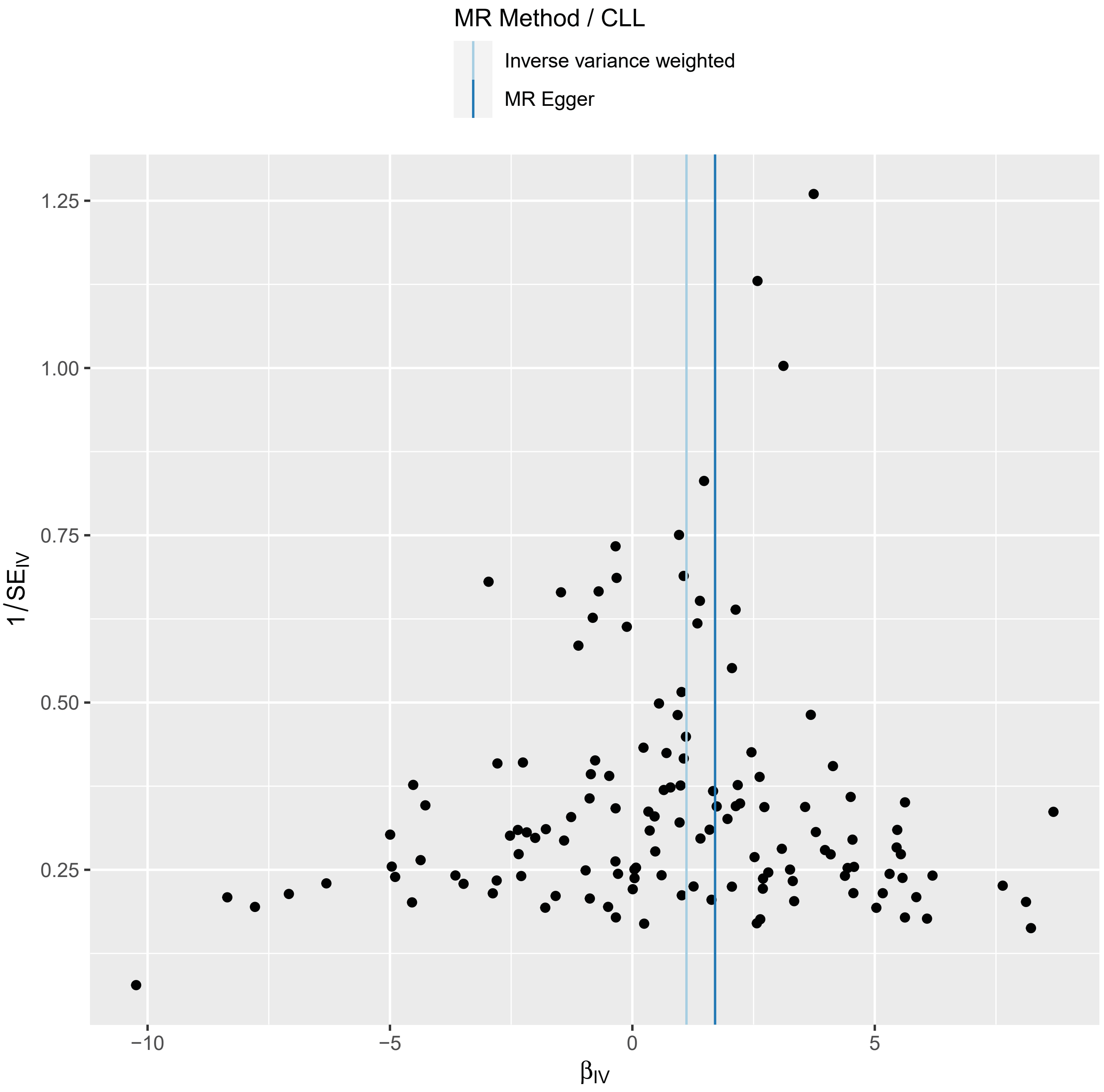
MR Method / CLL
Inverse variance weighted
MR Egger
1.25
1.00
0.75
0.50
0.25
1/SE_IV
−10
−5
0
5
β_IV

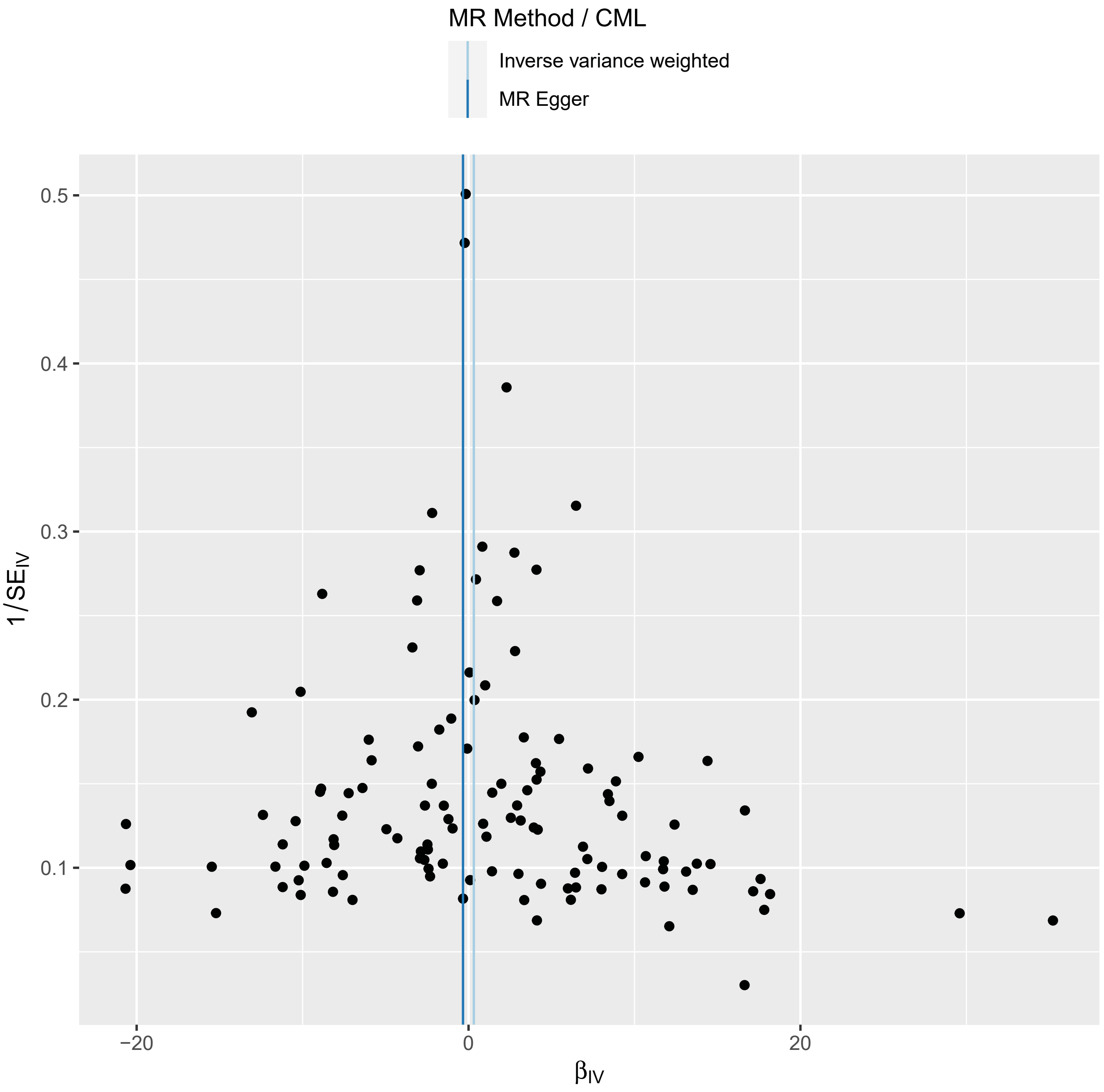
MR Method / CML
Inverse variance weighted
MR Egger
0.5
0.4
0.3
0.2
0.1
1/SE_IV
−20
0
20
β_IV

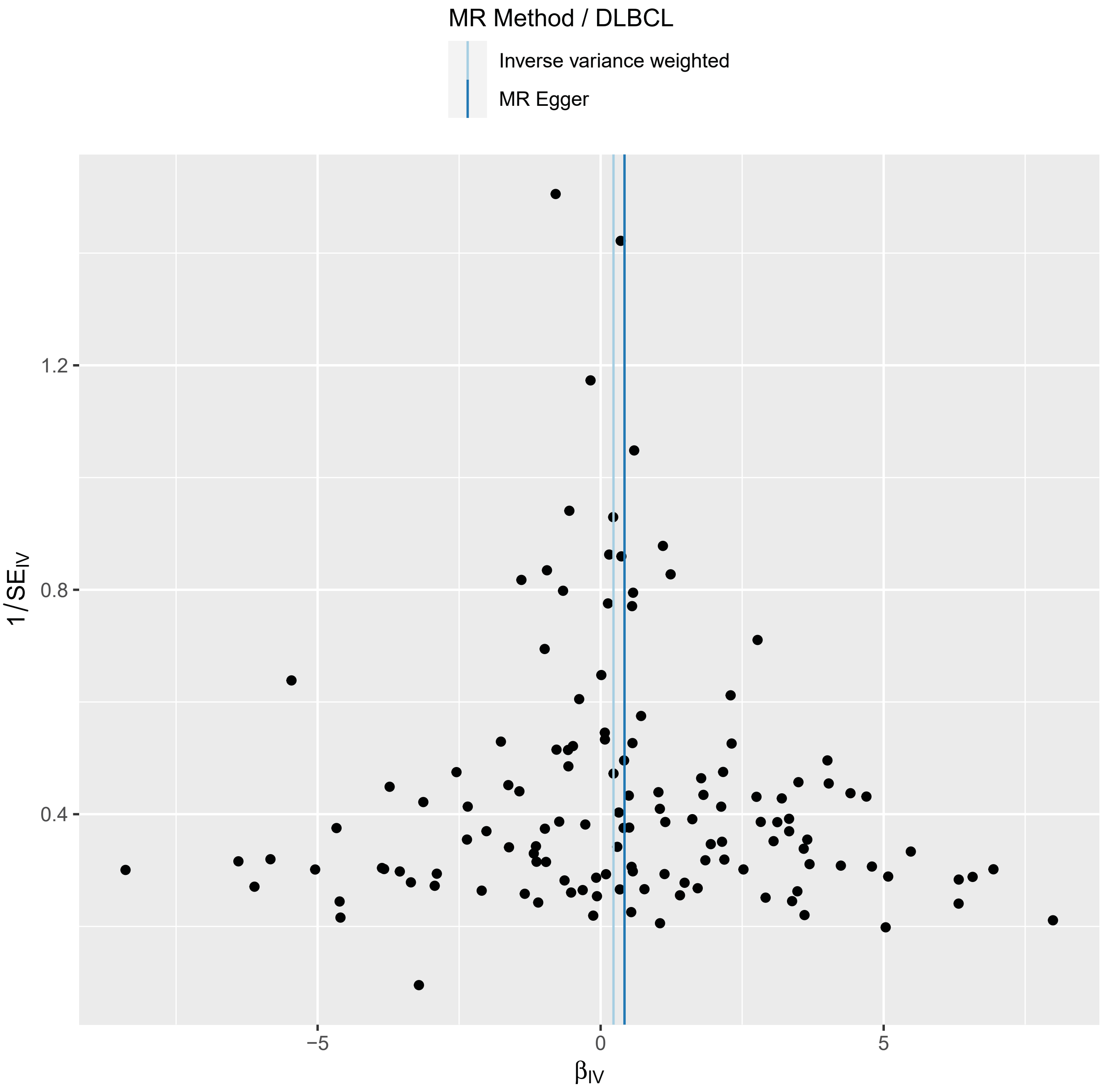

MR Method / DLBCL
Inverse variance weighted
MR Egger
1.2
0.8
0.4
1/SE_{IV}
−5
0
5
β_{IV}

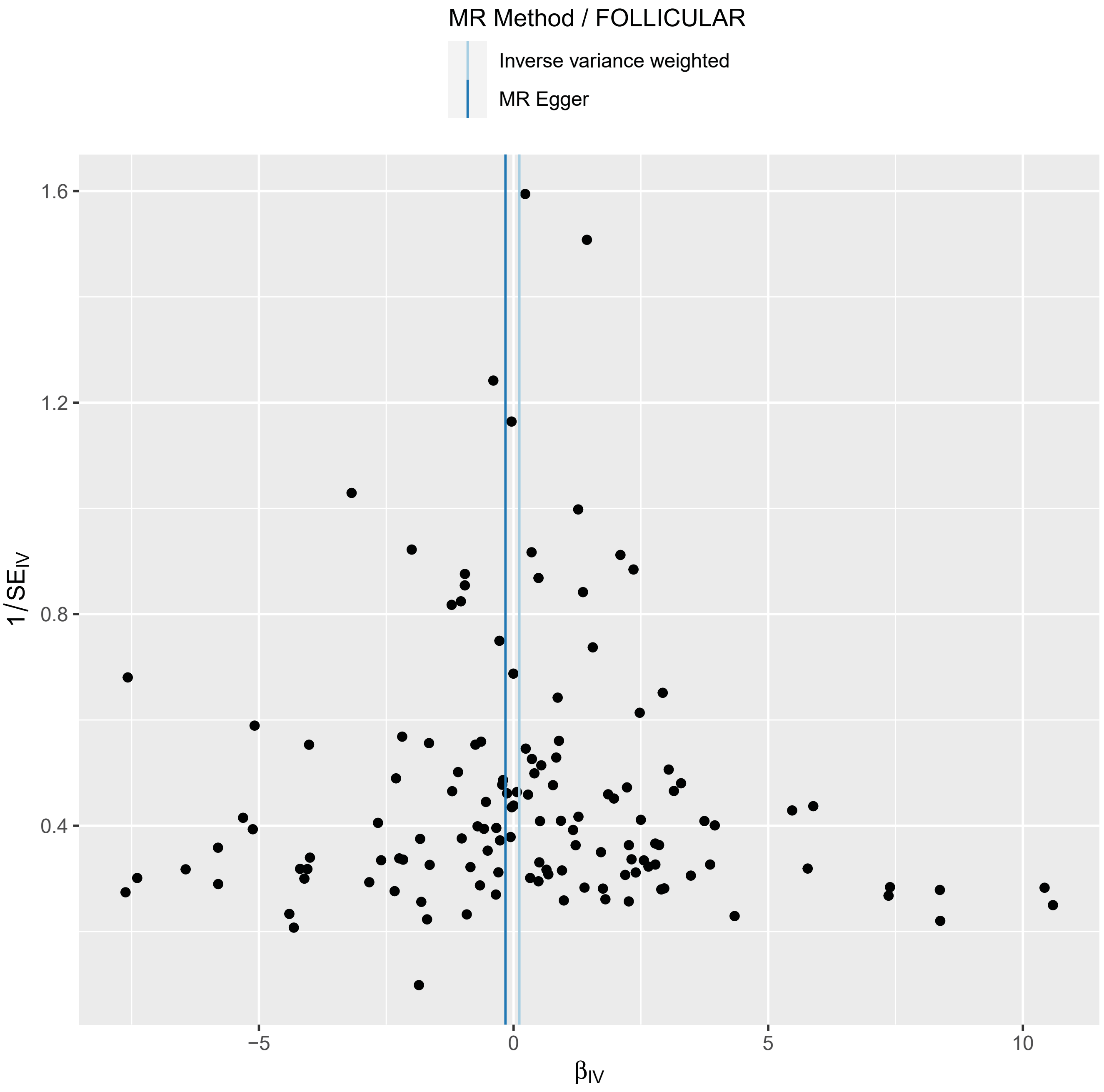

MR Method / FOLLICULAR
Inverse variance weighted
MR Egger
1.6
1.2
0.8
0.4
1/SE$_{IV}$
−5
0
5
10
β$_{IV}$

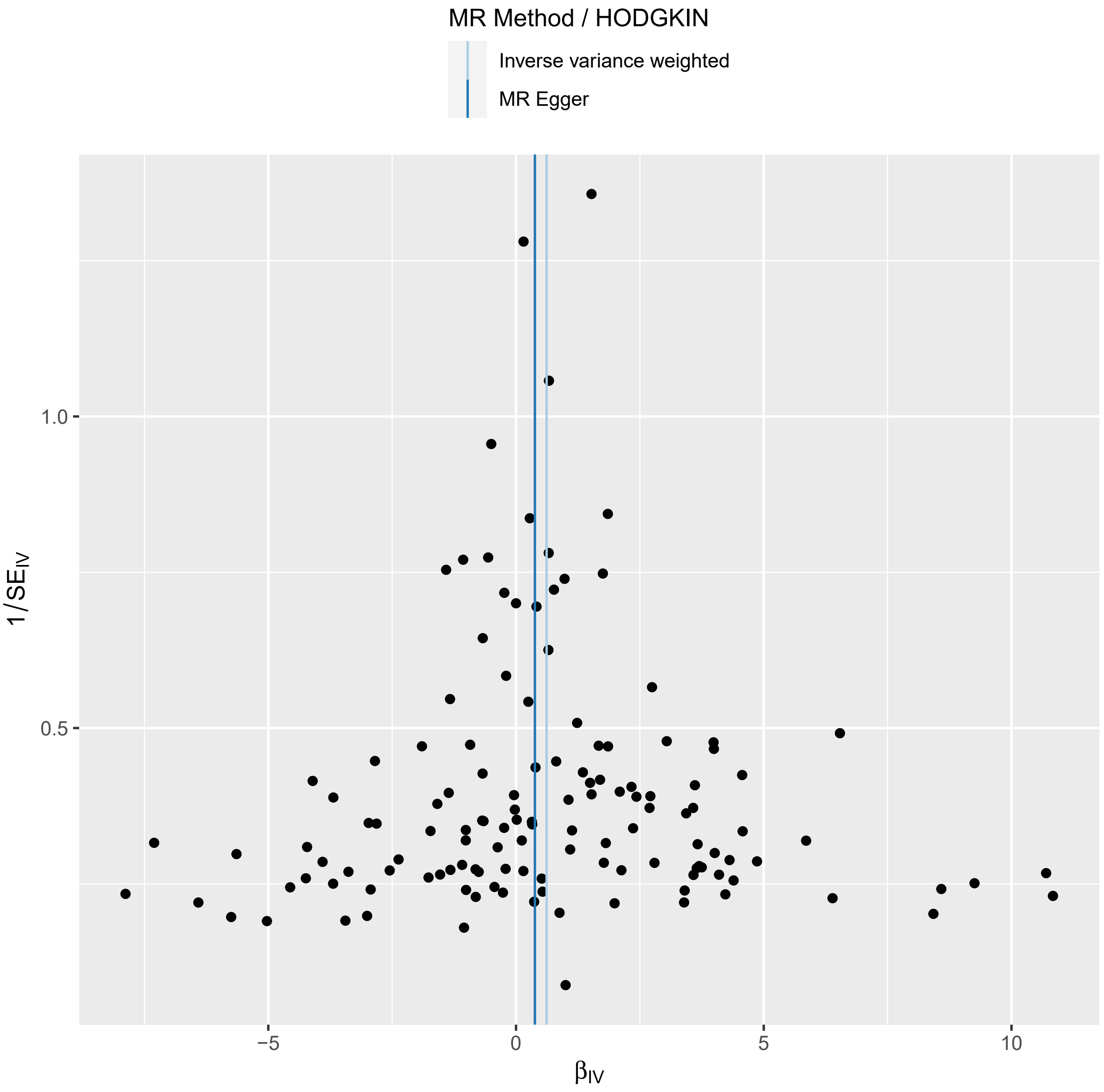

MR Method / HODGKIN
Inverse variance weighted
MR Egger
1.0
0.5
1/SE$_{IV}$
−5
0
5
10
β$_{IV}$

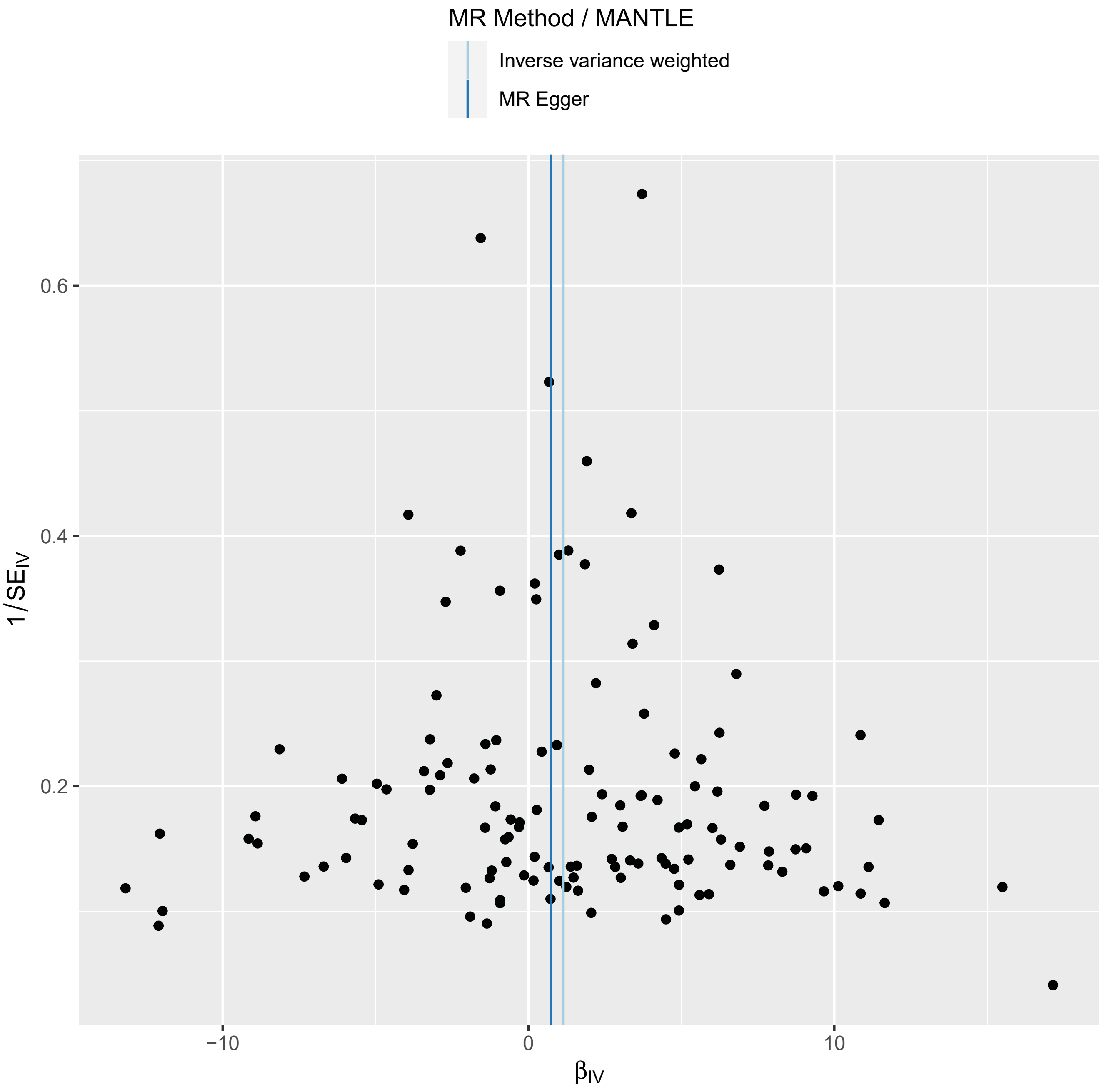
MR Method / MANTLE
Inverse variance weighted
MR Egger
0.6
0.4
0.2
1/SE_IV
−10
0
10
β_IV

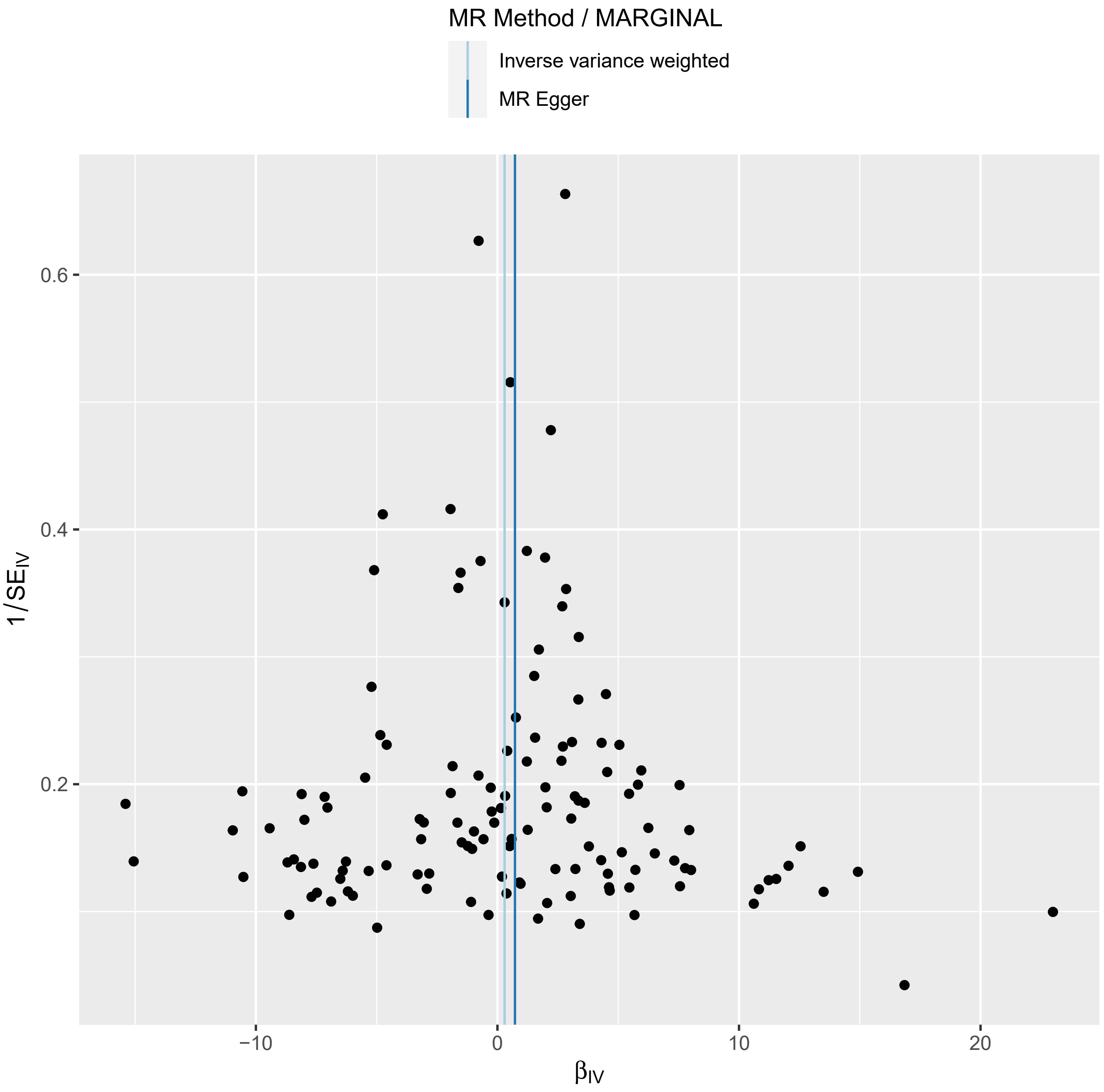

MR Method / MARGINAL
Inverse variance weighted
MR Egger
0.6
0.4
0.2
$1/SE_{IV}$
−10
0
10
20
β_{IV}

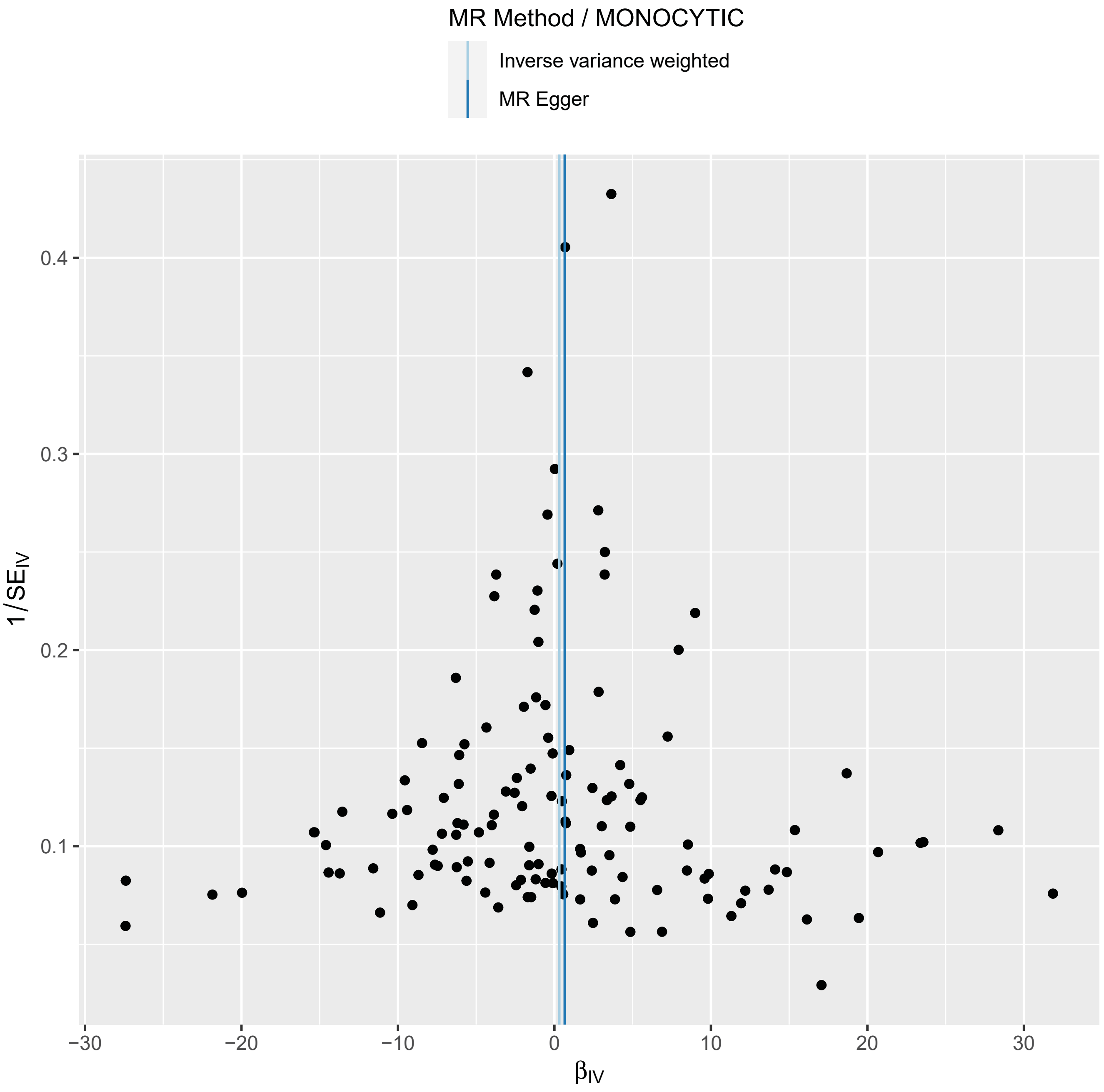

MR Method / MONOCYTIC
Inverse variance weighted
MR Egger
0.4
0.3
0.2
0.1
1/SE_IV
−30
−20
−10
0
10
20
30
β_IV

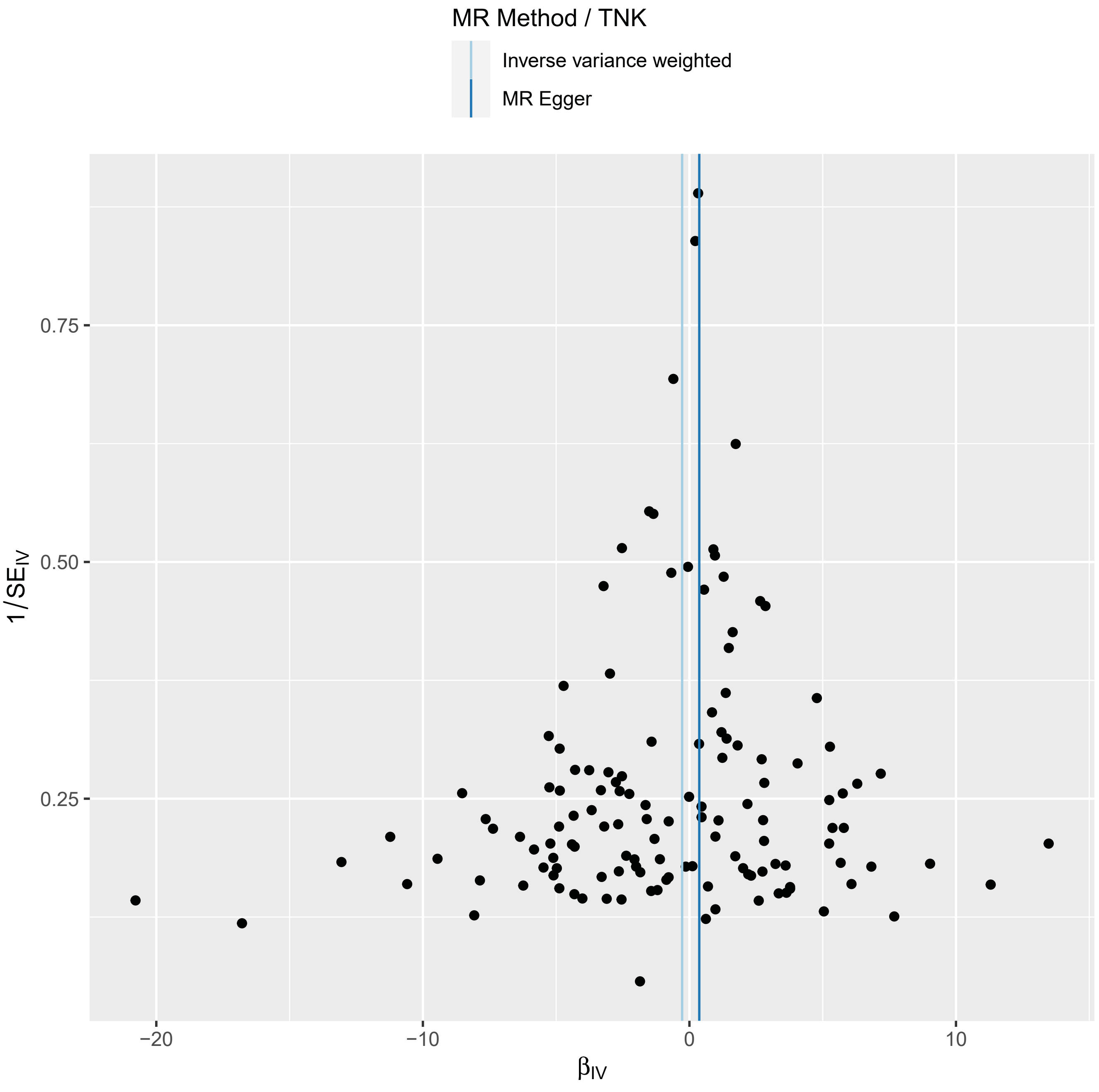

MR Method / TNK
Inverse variance weighted
MR Egger
1/SE_IV
0.75
0.50
0.25
−20
−10
0
10
β_IV
